# Breaking the cycles of violence with narrative exposure: Development and feasibility of NETfacts, a community-based intervention for populations living under continuous threat

**DOI:** 10.1371/journal.pone.0275421

**Published:** 2022-12-19

**Authors:** Sabine Schmitt, Katy Robjant, Thomas Elbert, Samuel Carleial, Anke Hoeffler, Amani Chibashimba, Harald Hinkel, Anke Koebach

**Affiliations:** 1 Department of Clinical and Neuropsychology, University of Konstanz, Konstanz, Germany; 2 Non-Governmental Organization Vivo International e.V., Konstanz, Germany; 3 Department of Politics and Public Administration, University of Konstanz, Konstanz, Germany; 4 Kigali, Rwanda; University of South Florida, UNITED STATES

## Abstract

**Background:**

Interpersonal violence damages mental health and frequently leads to aggressive defence strategies. If survivors are subsequently blamed for the events, both consequences worsen. Stigma flourishes, especially when survivors are silenced so that details of the trauma remain unknown. Breaking the secrecy both at the individual and collective level is key to enable the healing and reconciliation of individuals and communities living under continuous threat.

**Method:**

The *NETfacts health system* is a stepped care model with three components: (1) *Narrative Exposure Therapy* (NET), an evidence-based trauma therapy that includes survivor testimony (2) *NET for Forensic Offender Rehabilitation* (FORNET) acknowledges that perpetrators are frequently also victims and assists in reducing aggression and the attraction to violence, and (3) a community intervention disseminating and discussing *Facts derived from NET* treatment (NETfacts) to challenge the collective avoidance of atrocities and other traumatic material. The intervention was piloted in a community with 497 adult residents in Eastern Democratic Republic of Congo. The willingness of clients to consent to sharing their anonymised testimonies (with a focus on sexual violence survivors and ex-combatants) was investigated, together with other components of feasibility including security and clinical safety, extent of support of respected local authorities and participation rates. As secondary outcomes, clinical and social measures were assessed before and post NETfacts among 200 village residents of whom 160 self-enrolled and 40 had not participated in any form of treatment.

**Results:**

Implementation was feasible with 248 clients from a partner project giving consent to use their testimonies and high support of respected local authorities and participation rates (56% of residents self-enrolled in NETfacts). Immediate beneficial effects were shown for posttraumatic stress and rejection of rape myths among NETfacts participants who experienced multiple traumatic events in their own past. Attitudes towards ex-combatants improved and the perceived lack of social acknowledgement after trauma increased independent from participation. No significant change was observed for depressive symptoms.

**Conclusion:**

NETfacts is a feasible and promising approach to challenge the culture of secrecy surrounding trauma, suppression and social exclusion. Long term effectiveness requires further evaluation.

## Introduction

Interpersonal violence has been a global problem since time immemorial with conflict areas remaining the evident hotspots. Consequences for survivors include mental disorders such as posttraumatic stress disorder (PTSD), anxiety and depression [1, 2] as well as aggressive behaviors both among traumatized victims and perpetrators [3]. After the trauma, insult often adds to injury via stigma and social rejection, which not only further victimizes survivors [4–10] but can also trigger violent responses towards the rejecters [11]. At the core of these individual and societal drivers of violence is the avoidance of engaging with traumatic material and the human tendency to separate into social groups. The highly prevalent avoidance in traumatized communities breeds a culture of secrecy and prevents the integration of a coherent and accurate narrative of the traumatic event into the autobiographic and the collective memory, resulting in psychopathological responses among survivors and discriminatory norms and behaviour in their social realm [12].

In the context of post-conflict rehabilitation, prior interventions have either focussed on the individual (e.g. treating survivors’ mental health [13]) or created a forum for truth telling and education about traumatic experiences (e.g., Truth and Reconciliation Commissions [14]; radio or television edutainment [15]) to collectively process the past. This article outlines the rationale, development and feasibility of the *NETfacts health system* which integrates individual and community based treatment. NETfacts is a stepped care model combining (a) evidence based, narrative trauma therapy for survivors with posttraumatic stress symptoms to restore mental health and document the most important experiences with (b) a community based intervention that highlights the most relevant facts evident from the accumulation of narratives that are disseminated and discussed within community meetings in an anonymised, collectively relevant way in order to break the avoidance and restore the collective identity of the community.

### Trauma and the individual

Memories are formed as associative networks organized in sequences of time and location for retrieval. However, the mnesic storage of *traumatic* experiences is substantially different. Implicit details such as peritraumatic cognitions, emotions, interoceptive and sensory elements (‘hot’ memories) can be detached from their spatio-temporal context (explicit ‘cold’ memories [16, 17]) and ‘burned’ unconsolidated into the implicit memory [16, 18, 19], leading to perceptions of omnipresent threat, re-experiencing of the trauma (intrusions/flashbacks), hyperarousal and hypervigilance [20]. While surviving violence involves a negative valence (action disposition: flight, submission)–and has been associated with PTSD, anxiety, depression, and reactive aggression–perpetration of violence by contrast can contain an ambivalent or positive valence (action disposition: fight, domination) and has been linked to antisocial personality disorder, psychopathy and an ‘appetite’ for aggression [3]. The opposing valences of the underlying associative *fear* and *hunting* networks prevent their fusion [21, 22] and create a bi-cyclic structure of mental health problems and behavioral consequences following trauma and perpetration [3].

Survivors’ communication about their trauma is often inhibited, with manifold reasons including (a) the avoidance of being reminded of past horrors, which prevents the reappraisal of meaning [23], (b) an impaired verbal accessibility of the memory [24–26], especially if dissociative responding was part of the experience [27, 28], or (c) selective mutism due to trauma-related social affects such as shame [29] and guilt [30], fear of social suppression [31] and political constraints [32], or to prevent future victimization from the perpetrators [33, 34]. Importantly, this speechlessness cannot only prevent survivors from seeking professional help (e.g., doctors, therapists, lawyers) but also leads to secrecy surrounding trauma in the social realm.

### Trauma and the community

Survivors’ avoidance to engage with the traumatic material can develop momentum and successively converge into collective avoidance. Especially in (post) conflict areas where the threat is omnipresent, people have the conflicting experiences of both learned helplessness and the urge to restore safety. This cognitive dissonance can be resolved by exaggerating individual characteristics as causes of trauma [35] as this re-establishes the belief in a just world [36–39] where experience of trauma remains controllable. Especially in situations where survivor testimonies are lacking due to individual avoidance of communicating about their trauma, these socially constructed ‘explanations’ can become collectively accepted and established as truth, despite neglecting the actual facts [40–42]. If a critical mass in the community adopts this coping strategy, norms that institutionalize this ‘othering’ may emerge and endorse discriminatory behavior towards survivors [43].

Where survivors are blamed, stigmatised or excluded their social pain increases [4–10] and mental health problems worsen [44]. This also suppresses survivor voices [31] which further reduces the community’s exposure to the actual testimonies. The culture of secrecy surrounding trauma is therefore maintained. Paradoxically, these dynamics increase aggression among the rejected [11] and protect the perpetrators who likely continue to resort to violence [21].

Thus, whereas a fragmented autobiographical memory underpins mental disorders and (reactive/appetitive) aggressive behaviour among the traumatized, an incomplete collective memory can promote discriminatory norms and behaviors in the community which further victimizes survivors and triggers more violence. It prevents survivors from speaking out and communities from offering support for recovery, which ultimately decreases functionality of the whole community especially in conflict areas where violence remains part of everyday life. Breaking the secrecy and creating a coherent and accurate narrative of the traumatic experience both at the individual and collective level is key to healing and reconciling populations living under continuous threat. Importantly, avoidance of engaging with the traumatic material is pathological at the survivor level and pseudo-pathological at the community level and therefore, call for different treatment approaches [12].

### The NETfacts health system

**Fig 1:**
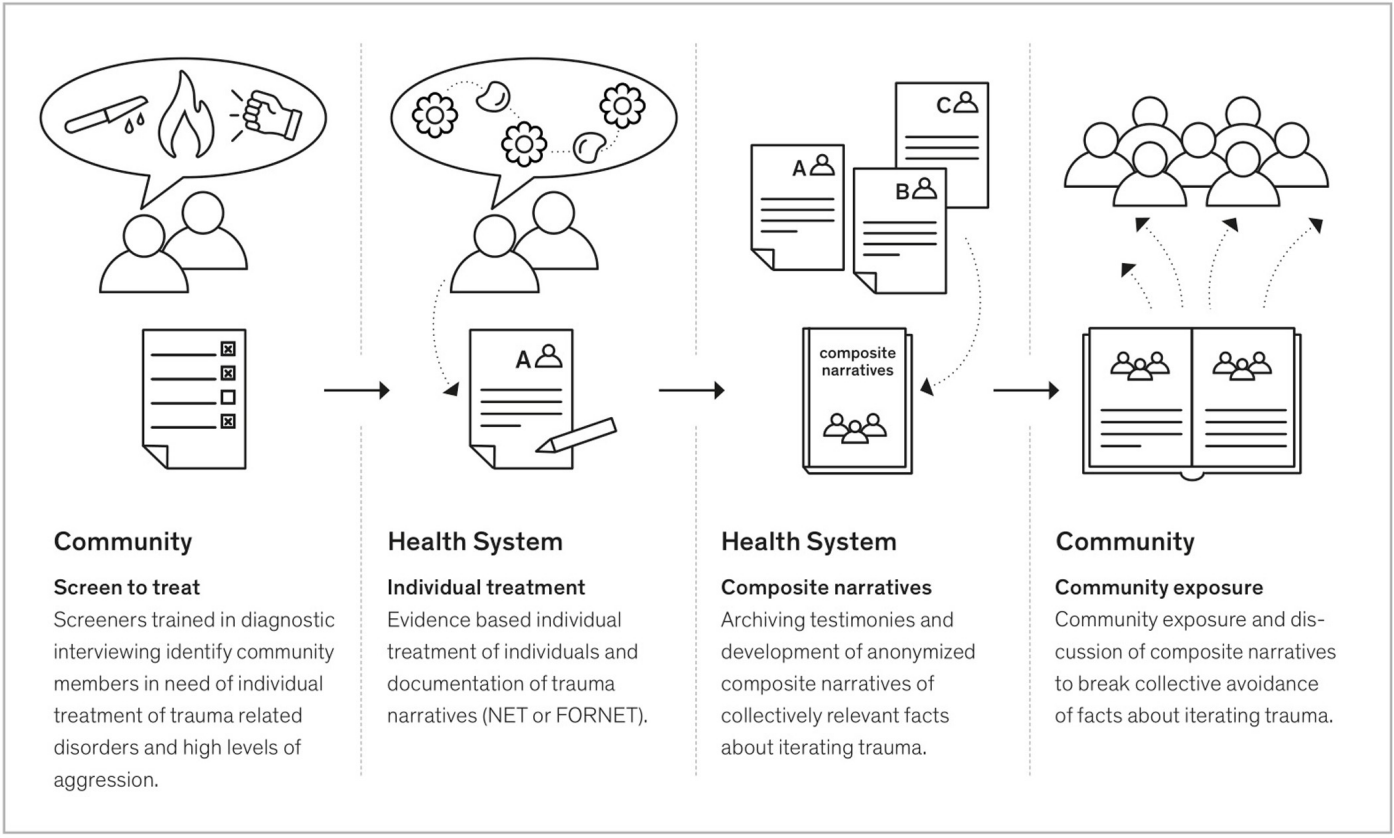
The NETfacts health system.

The *NETfacts health system* was developed as a stepped care model with high intensity trauma therapy for survivors who present with clinically relevant symptoms of trauma-related mental disorders and a low intensity intervention for the traumatized community. As shown in Fig 1, this includes (a) a screen-to-treat approach to identify and refer trauma survivors with clinically relevant symptoms (either PTSD diagnosis or high appetitive aggression) to therapists in the local health system who are trained in *Narrative Exposure Therapy*, NET [45] or its adaptation for former perpetrators, *NET for Forensic Offender Rehabilitation*, FORNET [46, 47]–hereafter, ‘NET’ is utilized to refer to both NET and FORNET–and (b) a community intervention that disseminates and discusses *Facts derived from NET* treatment, NETfacts, and includes the offer of singular exposure sessions for subclinical community members to enhance group resilience and increase ownership of the shared facts [48]. This combined approach aims to treat those with trauma and / or aggression while also encouraging disclosure and dialogue in order to allow a more coherent and accurate narrative of past traumatic experiences both in the autobiographical and collective memory–an important step towards individual healing and a sense of communion within communities.

The primary aim of this study is to pilot NETfacts in a (post) conflict setting, the Eastern Democratic Republic of Congo (DRC), firstly to test its feasibility and secondly, to provide initial insights into immediate effects on clinical and social measures.

## Methods and materials

### Study setting

The DRC counts as one of the most politically fragile and insecure regions in the world [49]. Multiple armed groups contribute to the ongoing violence [50, 51]. Armed groups that were or still are active in the intervention area are the Alliance of Democratic Forces for the Liberation of Congo (AFDL) in 1996 during the first Congo war, the National Congress for the Defense of the People (CNDP) in 2004 in its retreat from the occupation of Bukavu city, where it had become feared for the war crimes committed, the Alliance of Patriots for a Free and Sovereign Congo (APCLS) Hunde tribal militia (often referred to as Mai-Mai), besides the National Army Armed Forces of the DRC (FARDC) and Congolese National Police (PNC) [52]. The narratives of sexual violence survivors [53, 54] and of those who have been abducted by armed groups and turned into fighters [52, 55] reveal the true horrors of the conflict. Both rape survivors [56] and ex-combatants [57, 58] face high levels of stigma within communities and research has shown that a combination of others’ rejective attitudes and recently experienced stigma predicts rape survivors’ mental health [59] and ex-combatants’ ongoing violence [60].

### Procedure

**Fig 2:**
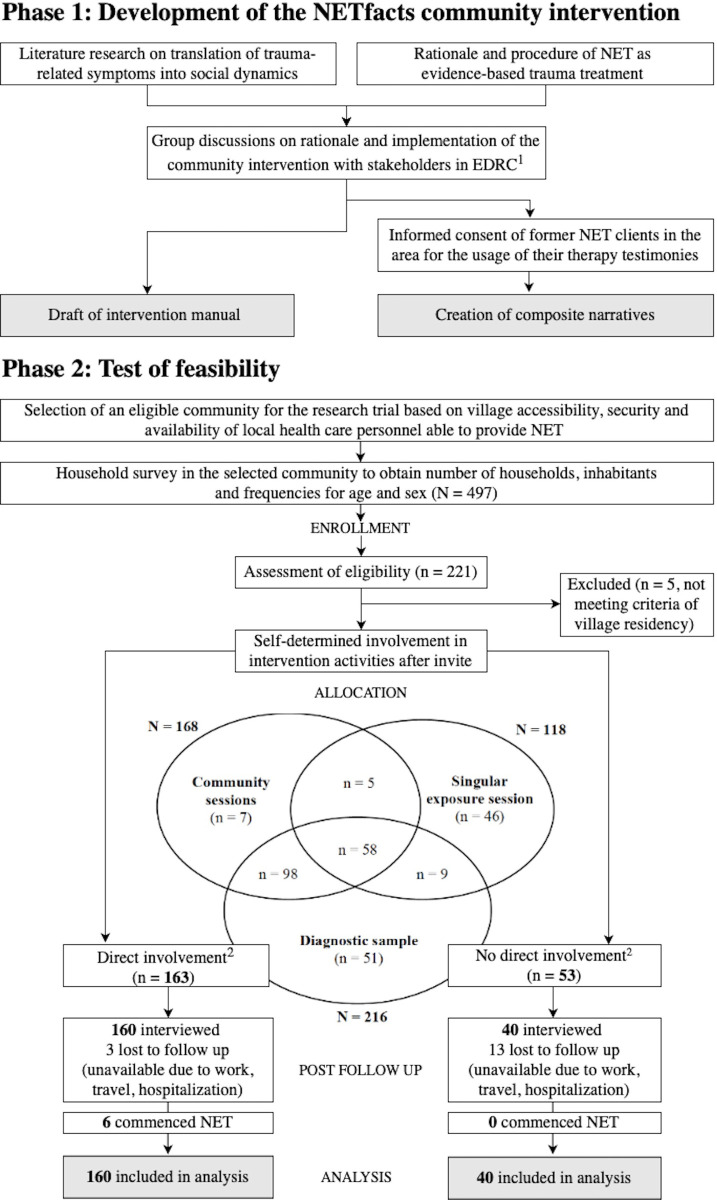
Flow of development and feasibility of NETfacts. Note. ^1^ for more information see S1 Table, ^2^ direct involvement is defined as either commencing NET, singular exposure session, or participation in at least one community meeting including composite narratives, whereas no direct involvement means individuals who were not directly involved but lived in the same community (and so may have encountered spill-over effects).

The study was implemented in a rural community located 1.5 hours away from Goma, North Kivu, in the Eastern DRC from June to December 2018. Diagnostic interviews were carried out in the participants’ homes, trauma therapy (NET), in the health centre of the neighbour village (approximately 15 minutes by foot), and the community intervention (NETfacts) in the village’s church as it was the most spacious available facility. Fig 2 shows the flow of development and pilot of NETfacts. The study was approved by the ethics commission of the University of Konstanz (IRB statement 31/2016) and the Fond Social of the DRC.

#### Selection of the community

First, official permission required for working in the village was obtained by the territory administrator and the chief of the grouping (county). Then, two communities were approached through individuals with respected local authority (e.g., head of the village, ‘notable’ community members, representatives of the women, youths or church) and compared in regard to accessibility, security and availability of a nearby health centre with personnel trained in NET. In a partner project implemented since 2016, Congolese nurses in health centers and members of community-based or national non-governmental organizations have been trained to deliver NET. After a 3-week training, they provided therapy in remote areas in North and South Kivu, Eastern DRC, supervised via telephone and field missions by experienced local counselors from our team and a British licensed clinical psychologist on site (second author). In June 2018, one of the two communities was chosen.

Since there was no reliable census data, a pre-survey was conducted to assess the number of households and adult inhabitants including frequencies for different sex and age groups (female vs male, 16 to 36 yrs vs 37 to 57 yrs vs over 57 yrs, see S1 Table). The sample size representative in sex and age was calculated for the 497 adult residents based on estimates obtained in this pre-survey, included a 5% margin of error and a 95% confidence interval and yielded at *N* = 216. This estimation was used as starting point for the sampling procedure (see next paragraph).

#### Selection of community members for diagnostic interviews

Inclusion criteria for diagnostic interviews were village residency and a minimum age of 16 years. Trained local psychologists (Bachelor or Master University degree) carried out clinical interviews under continuous supervision by three of the authors on site. Door-to-door visits were coordinated by a member of our team in cooperation with the village chief or one of his representatives. Interviewers randomly selected one person per household while controlling for sex and age groups. Each interviewer obtained an envelope with folded papers which were labelled with the required combinations (e.g., “Man, 16 to 36 yrs”). Where the household gave no match or the selected participant was unavailable, interviewers were instructed to draw another paper. Lack of availability was rare and mostly due to work or school attendance. If an eligible person lived in the household and was temporarily absent (but generally available on other days), an appointment was arranged. If more than one eligible person lived in the household, the interviewer offered folded papers (all blank except one labelled with a black cross) and interviewed the person who picked the mark. Towards the end of approaching all households, young men appeared to be underrepresented. To obtain age and sex representativeness, individuals with respected local authority assisted in recruiting thirty-seven individuals non-randomly (17% of the sample).

#### Implementation

Clinical interviews were carried out in July 2018 within participant’s homes and lasted between 1.5 and 2.5 hours. Light refreshment was provided. After randomization and confirmation of availability, participants were informed about the purpose, content and process of the study. No one declined participation and all signed a written informed consent. In case of illiteracy, interviewers read out the informed consent and obtained a fingerprint in place of the signature. The fact that participation was voluntary was repeatedly emphasised.

Participants who presented with clinically relevant symptoms (i.e., either PTSD diagnosis or high appetitive aggression) were referred to NET counsellors in the local health system. Before the first NETfacts session, individuals with respected local authority were invited to a group discussion on collectively relevant events in the community’s history that are to be expected to be mentioned in the big gathering. In the meantime, all diagnostic interviewees were invited to the community intervention at the local church in August 2018. For this purpose, invitation letters were distributed which indicated the date of the first meeting and a short description of the intervention’s content. A family member or friend was permitted to attend if the interviewee was unavailable. Therefore, diagnostic interviewees either self-enrolled in NETfacts (direct involvement) or decided against participation (no direct involvement, cf. Fig 2). After the last NETfacts meeting in September 2018, two group discussions with seven women and eight men from the diagnostic sample were conducted to assess comprehension about the NETfacts intervention (e.g., lifeline symbols, content and discussions of survivor testimonies). Participants were preselected with regard to age, experience or witness of sexual violence, history of armed group involvement, commencement of NET and, importantly, involvement in NETfacts to ensure a variety of different experiences with the intervention components. Discussions lasted on average 30 minutes and were audio-recorded with informed consent. A financial compensation for pre-, peri- and post NETfacts session participation was limited to respectively 1.000 CFC (about 0.60$).

Post follow up interviews were carried out immediately after the last NETfacts community session.

### Participants

The completer sample at post follow up was 200 individuals (of 221 interviewees at baseline, Fig 2). Participants were between 16 and 93 years old (median 35) and in 54% of the cases female (*n* = 107). All were Congolese with different native languages (*n* = 122, 61%, Kihunde, *n* = 46, 23%, Kiswahili and *n* = 32, 16%, others). Less than half of participants were able to read and write (*n* = 90, 45%) with a median of three years of formal school education (range: 0 to 12 years) and 59% (*n* = 127) were in a relationship. Almost half of participants (*n* = 86, 43%) were born in the village; among the others, 53% (*n* = 59) reported security problems in their home villages as reason for migration. Financial wealth was rated as low on a 5-point Likert scale with higher values indicating more prosperity (*Mdn* = 2, range: 1 to 5).

### Intervention

The NETfacts health system includes a screen-to-treat approach to refer trauma survivors with clinically relevant symptoms to evidence based therapy (NET) and a community-based intervention (NETfacts, cf. Fig 1).

#### Individual trauma therapy (NET)

NET [45] has evolved as one of the leading therapeutic approaches especially after experience of multiple trauma where each tramatic event ’builds’ on the burden of prior trauma and further increases the risk of PTSD (‘building block effect’ [61]). Research has shown its effectiveness even in insecure and conflict settings [62] including local counsellors without years of academic training [63–65]. NET’s adaptation for traumatized perpetrators, FORNET [46, 47], has shown to be effective in reducing PTSD, depression, appetitive and ongoing aggression [64, 66].

In NET, after a comprehensive diagnostic assessment and psychoeducation, the client lays out, in chronological order and guided by the therapist, his or her major life experiences (‘lifeline’). Time is symbolised along the course of a rope (beginning at birth and ending with the unknown future, which is represented as coiled-up part of the rope), flowers to represent major positive events, stones for major negative and traumatic events and sticks for major violent perpetration which is frequently associated with a mixture of reactive and appetitive aggression and, therefore, may have an ambivalent valence. The lifeline is used as basis to plan and structure the following exposure sessions in which the client is guided to narrate and remember affect, thoughts and physiological responding elicited by the most traumatic events (including severe perpetration). The therapist ensures coherence of the recalled details (sensory perceptions, cognitions, emotions and body feelings) and strongly emphasises the context in which the event happened (time and location). Sensory, cognitive, emotional and interoceptive information of the present and past are continuously contrasted during the exposure. In this way, traumatic memories are located in the past time and location and clients learn to recall the event without fear or otherwise high emotional arousal. Successful treatments for PTSD have further shown a positive effect on functionality in work and social roles [67], externalizing behaviour problems [68–70] and to rebuild shattered beliefs about the world and increase hopefulness for the future [13]. NET ends with the exploration of wishes which are symbolized as flowers on the coiled-up part of the rope that represents the unknown future. Throughout the treatment, the therapist creates a written record of the autobiographical narrative which can be handed over to the client at the end of the treatment. These NET testimonies serve as basis for the NETfacts community intervention.

#### Community-based intervention (NETfacts)

The core of NETfacts involves the sharing of the avoided facts about violence in the community, unknown by many. In this study, anonymized versions of individual NET testimonies were used to create *composite narratives* that describe the typical experience of an iterating and therefore collectively relevant traumatic event from the survivors’ perspective, and in particular its psychic components such as trauma-related sensory, cognitive, emotional and interoceptive reactions. To obtain composite narratives, we selected the most frequent themes that emerged in the treatments based on the available testimonies. Secondly, the *facts* about survivors’ sensory, cognitive, emotional and interoceptive reactions as well as basic information about time and location were merged into one composite narrative per traumatic theme. Composite narratives were developed for three major categories: (1) village attack by an armed group, (2) rape-related narratives (including: rape by armed men; stigmatization after rape; forced marriage ritual in the community including rape), and (3) combat-related narratives (including: abduction into an armed group; experiences as combatant including torture, witness murder of fellow recruits as punishment for escape attempt, non-intended killing of civilian instead of enemy soldier, and killing of civilian forced by commander, escape and stigmatization in civilian life). All composite narratives were developed and reviewed by the authors to ensure coherence and complete anonymization. Ten group discussions with rape survivors, ex-combatants, locals without a history of rape or armed group involvement as well as local experts on survivors’ trauma (therapists and members of community-based or non-governmental organizations) provided initial insights and a local perspective of potential risks and opportunities for disseminating and discussing NET testimonies into communities (S2 Table). The concept of sharing NET testimonies was supported in all discussions as long as anonymization was guaranteed and survivors contributing to composite narratives were from different villages than the NETfacts intervention villages. To enhance commitment, participants suggested involving a wide range of individuals with respected local authority and to discuss the narratives with the community after reading them out. Concerns were raised about the trustworthiness and confidentiality of community members if they would be trained and included as NET therapists.

The community based intervention contained four sessions with a two-week break after the first and one-day breaks between the second, third and fourth session. In the first session, the established symbols of NET were applied to create a *community lifeline*. To this end, participants were invited to identify major events relevant for the community’s history and lay them out along a rope in chronological order (to be aware of the main events to expect, respected local authorities were gathered to lay out this lifeline before the first NETfacts session). Each time after laying down a symbol for a collectively relevant event, participants were encouraged to name own experiences that happened during this event or experiences of family members or friends (without revealing their identity), to broadly describe them and then to place a symbol on the rope as part of the collective event. A village attack, for instance, represents a collective fearful or traumatic event but can contain diverse individual experiences such as running for life, witness of torture, being raped, being abducted into an armed group or, especially in case of combatants, being forced to kill or willingly killing another person. At the end of session one, all community members were offered the opportunity to process and narrate one of their own events in a *singular exposure session* during the upcoming two weeks before the second session. This offer was only available for those who had not been diagnosed with PTSD or presented with high appetitive aggression since any such cases were to be referred to a NET counsellor for a full treatment. Prior research has shown that NET with reduced sessions (four or five instead of eight to 12 sessions) can decelerate the building block effect of trauma [65, 71]. Singular exposure sessions were therefore included for the subclinical cohort. By reducing the trauma load, these sessions may prevent the development of clinically relevant PTSD in the event of further trauma exposure. In addition, processing an own traumatic event increases identification with the intervention and heightens the ownership of the facts that will be shared through composite narratives in the following sessions. Importantly, if posttraumatic stress decreases, associated symptoms may reside as well (e.g., feelings of hatred, anger, revenge, caveats to forgiveness and reconciliation). Therefore, participants may be more responsive to composite narratives and the ultimate goal of decreasing stigma and increasing social cohesion. During the two-weeks following the first session, composite narratives were selected from the collection that had been created previously. Traumatic themes that were relevant for the community were chosen, as indicated by (a) NET treatments (themes occurring in treatments that had commenced from the clients who had been referred into the local health system after the screening), (b) singular exposure sessions, and (c) the community lifeline. In three further meetings of which each began with a brief reminder of the events placed on the lifeline, the chosen composite narratives were read out and discussed (*community exposure*). Six NET counsellors were available to provide specialised support if participants seemed distressed, to ensure referral to professional care if needed and/or to follow up after the session. After listening to the composite narratives, participants were encouraged to share their own thoughts and emotional reactions. In two experiments, Harber, Podolski [72] showed that expressing emotional responses to others’ victimization can reduce victim blaming. Participants were then asked to anticipate the differing needs of the narrative’s protagonist after experiencing this event. Each discussion was finalised by reconciling the needs of the traumatized protagonist with those of the community. The last session was closed with collecting wishes for the community’s future, laid down as flowers on the coiled-up part of the lifeline. Facilitators ensured that the expressed wishes remained realistic as prior research showed that unrealistic fantasies about one’s future may enhance positive feelings in the short-term but increase depressive symptoms in the long-run [73]. Finally, a poster of the lifeline was handed over as commemoration of the community’s past. All sessions in this feasibility study were led by a British-licensed clinical psychologist (second author) and a Congolese NET counsellor (fourth author).

### Assessment

#### Primary outcome

Feasibility was evaluated based on the following criteria (1) before implementation: availability of informed consents provided by trauma survivors in a partner project to use their NET testimonies for the creation of composite narratives; official permission to operate in the area and community by formal and informal local authorities (chef of the “grouping” of villages), (2) during the implementation: security in the area evaluated based on continuous consultations with advisors from the United Nations and individuals with respected local authority from the village who were aware of more recent and minor safety risks and fluctuations within the community; clinical safety evaluated through the continuous presence of international clinical professionals experienced in mental health care; involvement in NETfacts operationalized as attendance and retention rates in community sessions; engagement during community sessions operationalized as observed attentive listening and active discussion; self-enrolment in singular exposure sessions and NET in the local health system after referral.

#### Secondary outcome

The diagnostic assessment set was administered as clinical interviews. All questionnaires were translated beforehand into the local language Kiswahili and back to English by two native speakers; differences were discussed with experts with experience in East African settings (second, sixth, and seventh author) to ensure accuracy. Demographics were assessed at baseline. Traumatic events, perpetration of violent acts, clinical measures (PTSD, depression) and social measures (disbelief in social reconstruction with ex-combatants, attitudes and beliefs about survivors of sexual violence, rape myths acceptance, and perceived lack of social acknowledgment as trauma survivor) were assessed at baseline and post follow up.

*Traumatic events and perpetration of violent acts*. Exposure to trauma was assessed with a short version of the Threats to Human Life Scale (THL; scale can be obtained from the authors; [74]). The 41-item checklist assesses threats to physical integrity (18 items), social integrity (8 items) and perpetration of violent acts (15 items) in the last three months (recent) and historically (lifetime; answer categories yes/no). Each item refers to a specific event type (e.g., suffocation, social exclusion, physical fighting). Further, the perpetrator of recent exposure to threats to physical integrity were assessed (i.e., ‘by family member/s or person/s of trust’, ‘by community member/s’, ‘by stranger/s or organised violence’, or ‘none manmade’). The subscale threats to social integrity was adapted by asking rape survivors during the diagnostic interview if they believe that they experienced the event because of their history of sexual violence or armed group involvement. For recent perpetration of violent acts, participants were asked to indicate the target/s (‘against family member/s or person/s of trust’, ‘against community member/s’, ‘against stranger/s or organised violence). One sum score was calculated for number of traumatic events based on the scales of threats to physical and social integrity and one sum score for perpetration of violent acts, both including time of exposure/perpetration (lifetime, recent) and its context (by/against family member/s or person/s of trust, community member/s, stranger/s or organised violence, none man-made; number of traumatic events, range: 0–104, perpetration of violent acts, range: 0–60). New traumatic events and violent perpetration after baseline were assessed at post follow up in three open questions asking whether the individual had experienced an event that threatened his/her physical or social integrity or s/he threatened the physical or social integrity of someone else; to this end, act based examples were provided.

*Clinical measures*. The PTSD Symptom Scale-Interview for DSM-5, PSS-I-5 [75, 76], was used to assess PTSD symptom severity. The scale consists of 20 items corresponding to symptoms defined in the PTSD clusters B to E of the DSM-5 [20] with response options ranging from 0 *(Not at all)* to 4 *(6 or more times a week/severe)*. Higher values of the sum score (range: 0–80) indicate more severe PTSD symptoms. The scale has been successfully used and validated in Africa [77] and in the DRC [64, 78]. In this study, internal consistency was good (α = .88) as was interrater reliability (IRR = .92).

Depression was measured with the 9-item Patient Health Questionnaire, PHQ-9 [79]. Each item corresponds to the DSM-5 symptom criteria for Major Depression and is rated on a scale from 0 *(Not at all)* to 3 (*Nearly every day*) referring to the last two weeks. Symptom severity is indicated by a sum score (range: 0–27). The instrument has been administered in African settings before [80] including DRC [65, 81] with excellent psychometric properties. Internal consistency (α = .85) and interrater reliability (IRR = .90) were good.

*Social measures*. The *Social Reconstruction Scale*, SoRS [82], evaluates the readiness to reconcile with former enemies and was developed to assess openness to social reconstruction between Croats and Serbs in post genocide Bosnia Herzegovina. The scale was adapted to measure readiness to reconcile with ex-combatants (e.g., “I am not ready to cooperate with ex-combatants even if my community asked me to do so.”). Two items were removed from the scale as they were non-applicable in this context. Nineteen items are rated from 0 *(Disagree strongly)* to 4 *(Agree strongly)*. A sum score is calculated to indicate the disbelief in social reconstruction with ex-combatants (range: 0–76). Whilst internal consistency was low (α = .46), interrater reliability was excellent (IRR = .94).

Rape myths acceptance was measured with an adapted version of the short *Illinois Rape Myths Acceptance Scale*, IRMA [83]. The original questionnaire comprised the four scales rated from 0 *(Disagree strongly)* to 4 *(Agree strongly)*: ‘Rape is a deviant event’, ‘He didn’t mean to’, ‘It wasn’t really rape’, and ‘She asked for it’. After internal discussions with local psychologists about how rape is misconceived in the local context, the scale ‘She owed him’ (4 items) was added to assess the belief that in some circumstances sex is owed to men (e.g., in exchange for goods or within marriage). This is in line with Buller, Pichon [84] who showed in a systematic review that sex is often expected in exchange for favors, and Tavrow, Withers [85] who specifically argue for including it in the assessment of rape myths. Higher sum scores for the 15-item scale (range: 0–60) indicate stronger acceptance of rape myths. Internal consistency was acceptable (α = .73) and interrater reliability excellent (IRR = .97).

Negative attitudes towards rape survivors and willingness to provide support was assessed with the *Attitudes and Beliefs towards Survivors of Sexual Violence Scale*, ABSV, developed in Kenya and DRC [86]. Participants indicated agreement with four statements on a 5-point Likert scale from 0 *(Disagree strongly)* to 4 *(Agree strongly)*. In line with the original publication [86], no sum score was calculated due to high heterogeneity of items. Interrater reliability was excellent (IRR = .94).

The subscale ‘*general disapproval’* of the *Social Acknowledgement Questionnaire*, SAQ [87] was used to assess the perceived lack of social acknowledgement as a trauma survivor. The subscale has shown to be strongly related with PTSD [88–90] and has been successfully used in previous studies in the region [64, 91]. Firstly, participants were asked if they had ever experienced a traumatic event after which they felt the need of social support. The five items were then rated in reference to this event from 0 *(I do not agree at all)* to 3 *(I completely agree)*. Higher values of the sum score indicate that participants perceive a more pronounced lack of social acknowledgement of the ordeal that they had survived (range: 0–15). Internal consistency was acceptable (α = .79) and interrater reliability excellent (IRR = .99).

### Statistical analyses of secondary outcomes

Statistical analyses were carried out in the completer sample (*N* = 200) using *R* version 4.0.0 [92] and *RStudio* version 1.2.5042 (https://www.rstudio.com). Subitems for calculation of sum scores were imputed using predictive mean matching as missing values per questionnaire maximum reached up to 5% of the sample [93]. To test the effects of NETfacts, generalized linear mixed models were carried out, GLMMs [94], for PSS-I, PHQ-9, SoRS, IRMA, and SAQ using the R packages lme4 [95] and glmmTMB [96]. Predictors were NETfacts involvement (direct vs indirect), assessment time point, and number of traumatic events. Direct involvement in NETfacts is defined as either commencing NET after referral (i.e., successful screen-to-treat approach), participation in a singular exposure session or enrolment in at least one community session including composite narratives. No direct involvement includes diagnostic interviewees who did not participate in NETfacts but may have been affected by spill-over effects as they lived in the same community. Number of traumatic events was included as predictor since own traumatization may influence the ability for perspective adoption with other survivor testimonies. Covariates were age, sex, years of education, perpetration of violent acts and new traumatic events experienced after baseline and included due to their potential association with clinical and social outcomes. To test the effect of NETfacts involvement over time and dependent on number of traumatic events, the three-term interaction NETfacts involvement vs time vs trauma and the two-term interaction NETfacts involvement vs time was tested.

To account for non-independence of measures, participant id and diagnostic interviewer were included as random effects. Since sum scores are count data, Poisson-distributed models were fitted for each outcome. For outcomes with excess zeros, models were fitted accounting for zero-inflation (zero-inflated Poisson and truncated Poisson Hurdle). Both zero-inflated Poisson and truncated Poisson Hurdle regressions are advantageous compared to Poisson and Negative Binomial regressions when fitting models with zero-inflated data. The two approaches model zero and count values separately in a logistic and a count model part. Whilst zero-inflated Poisson models separate *excess* zeros from observations with counts and occasional zeros, truncated Poisson Hurdle models treat zero and non-zero values as distinct categories [for an introduction and practical example see 97]. Hurdle models seem preferable especially for modeling mental health outcomes (PTSD, depression) under the assumption that symptoms emerge only if a certain threshold of mental strain is transgressed. This is likely true also for SAQ as this outcome depends on traumatic exposure and PTSD severity. The best model fit for each model was selected based on the Akaike’s Information Criterion (AIC) and other parameters (dispersion and residual fit). To assess model effects, the significance of the three-term interaction was first inspected. Where no significant three-term interaction was found, the model was refitted including the two-term interaction. Predictors were assessed for significance only if none of the tested interactions were significant. Non-significant interactions were excluded from the final models. In case of significant interaction effects, *post hoc* Tukey tests were carried out with the R package emmeans [98]. Models were plotted if either one of the interaction effects or the effect of NETfacts involvement or time were significant. For this purpose, model effects were first extracted using the R package ggeffects [99] and then plots were created with ggplot2 [100]. Paired sample t-tests were applied to evaluate changes on the four ABSV items for NETfacts involvement over time.

## Results

### Primary outcome

Before the implementation, the majority of clients who received NET (n = 248 of 259, 96%) in a partner project in Goma, North Kivu, DRC between 2016 and 2018 consented to contribute the anonymized version of their testimony for NETfacts to inform local communities about traumatic experiences from the survivors’ perspective. Each of the final composite narratives was based on an average of ten testimonies (n = 86 of the 248 trauma survivors). Official permission to operate in the area and community was granted by formal and informal representatives (chef of the grouping, village leader). Individuals with respected local authority consistently demonstrated support, including by assisting in outreach activities such as gathering village statistics, describing the intervention to the population, coordinating diagnostic interviews and community sessions. Before the first community session, they took part in a lifeline exercise in order that the moderators would be aware of the main events to expect.

During the implementation, security was successfully ensured based on the established security network. NET was well received by participants with clinically relevant symptoms who had been referred to therapists in the local health system after baseline (*N* = 36) and post follow up (*N* = 24). All received and finished the treatment within one year except three who had moved to another village (*n* = 57, 95%). On average NET was delivered in seven sessions (*SD* = 1.59, range 2–11) including two drop-outs respectively after two or four sessions. The average time period for session completion was 20 days (*SD* = 4.70, range 8–33). In addition, four residents approached one of our counsellors by themselves to ask for treatment. A total of 118 singular exposure sessions were delivered between the first and second community meeting. About half (*N* = 63, 53%) of these clients were individuals who participated in the first NETfacts meeting where the information about the offer to treatment was shared. In sum, by implementing NETfacts in the village trauma treatment was provided to 36% of adult residents (*n* = 179, of 497 inhabitants) with 12% (*n* = 61) receiving NET and 24% (*n* = 118) receiving a singular exposure session.

NETfacts community sessions lasted between 60 to 90 minutes. 168 individuals participated in at least one session which constitutes 78% of the invited individuals (*N* = 216). The majority of them (*n* = 117, 70%) attended all four meetings. Retention was as follows: 148 (74%) residents attended the first session, 123 (62%) the second session, 136 (68%) the third session, and 145 (73%) the fourth session. More members of the community expressed interest in participating, but due to space limitations this was not possible. Throughout all community sessions, moderators observed that most participants dedicated a high level of attention to the composite narratives that were read out. None of the participants were observed to exhibit extreme levels of distress such as flashbacks or dissociation, and neither were such experiences reported afterwards to the clinical professionals who were present in each session, suggesting clinical safety of the intervention. The read out of combatant-related composite narratives raised mixed opinions. In some cases, participants expressed empathy to the ordeal of the protagonist as victim. In other cases, anxiety and caveats to reintegration were emphasised owing to the fear of violent behaviour. The community finally agreed on considering the reintegration of ex-combatants, if they showed the willingness to adopt to non-violent civilian life and proved to be harmless in a gradual approach first by family members, then by peers and last by the broader community. Likewise, rape-related content elicited ambivalent reactions and some participants were ready to express prejudices which could then be discussed in the group. It was always possible to outweigh these isolated statements with integrative and reconciling opinions that were expressed by the majority of participants. The two feedback groups after the last community session revealed that participants had understood the lifeline and its symbols and remembered the content of most composite narratives. All indicated to have talked with non-attendees about the narratives.

In total, 278 residents were directly involved in at least one activity offered in NETfacts (i.e., referral to and commencing NET, singular exposure session, at least one community meeting including composite narratives), which constitutes 56% of adult inhabitants in the village (cf. Venn diagram in Fig 2, plus the four community members who approached one of our counsellors by themselves to request referral to NET).

### Secondary outcome

#### Sample description of compared groups

Individuals directly involved in NETfacts were younger (*t*(198) = -2.74, *p* = 0.007), more often female (*χ*²(1) = 22.26, *p* < 0.001) and less educated than those who were not directly involved (*t*(198) = -2.67, *p* = 0.008). There was no difference in partnership and immigration status or wealth (*p* > .05). Participants reported a median of eleven traumatic events (range: 1–30), whereby those directly involved in NETfacts indicated more events than those with no direct involvement (*t*(198) = -1.97, *p =* 0.050). Twenty-nine (15%) participants reported an incident of sexual violence and eight (4%) past involvement in an armed group. Twenty-one of the rape survivors (72%) indicated that they had experienced at least one social threat because of their rape history, whereas four of the ex-combatants (80%, *n* = 2 missing) reported the same in regard to their history of being in an armed group.

#### Clinical outcomes

**Fig 3:**
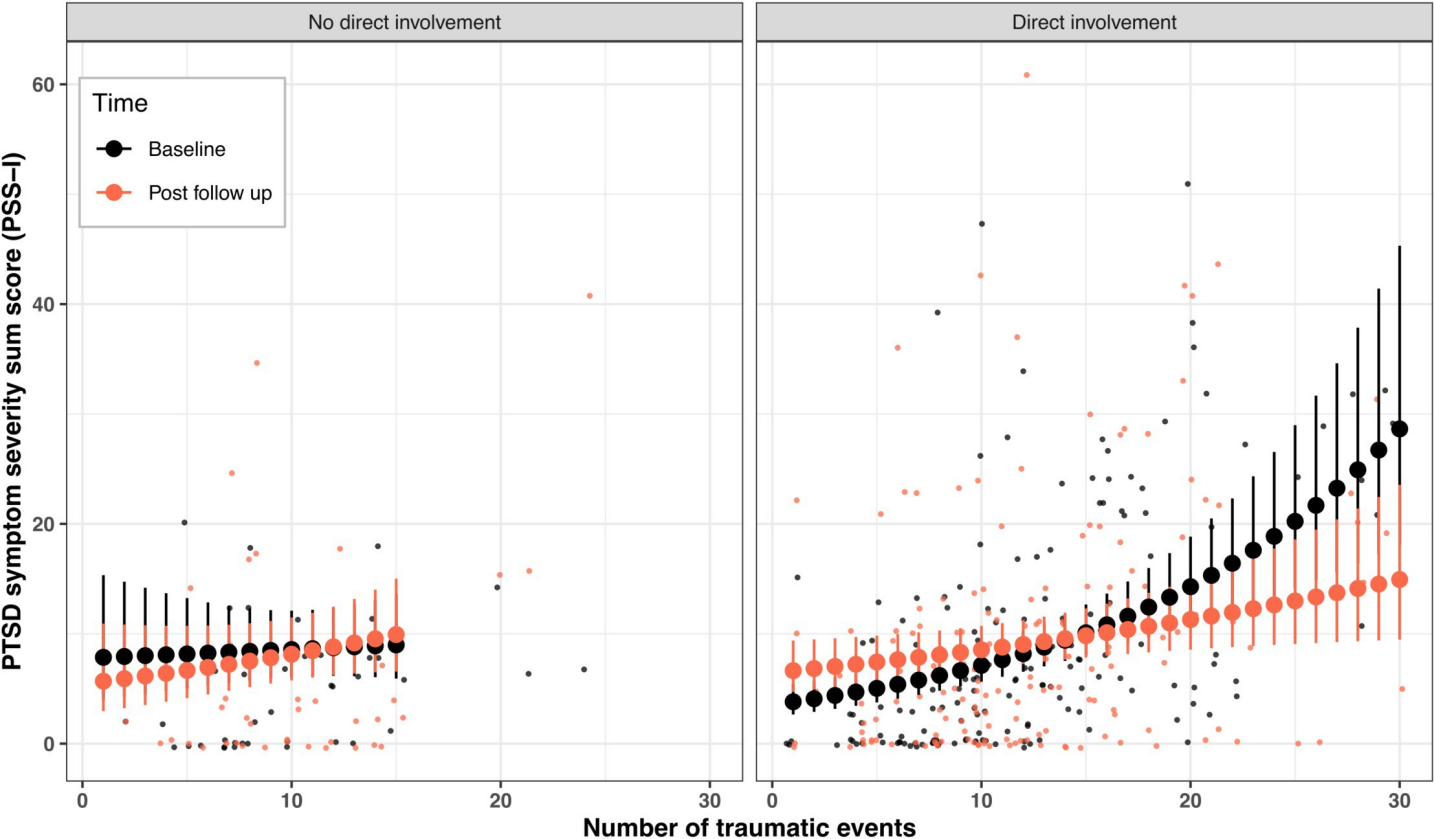
Significant interaction effect of the number of traumatic events among participants with no direct vs direct involvement in NETfacts from baseline to post follow up (time marked in color) on PTSD symptom severity. Small dots represent real individual observations, whereas large dots and error bars show mean estimates with 95% confidence intervals produced by the GLMM. Large dots and error bars for no direct involvement are presented up to 15 traumatic events due to small sample size.

GLMMs for PTSD and depression are summarized in Table 1. A significant three-term interaction was found for PTSD symptoms. Participants directly involved in NETfacts and with ≥ 18 traumatic events showed a flattened curve of the building block effect at post follow up. Participants with no direct involvement in NETfacts revealed an opposite trend (Fig 3). Of the control variables, experiencing at least one new traumatic event since baseline was associated with a higher PSS-I sum scores (*ß* = .22, SE = .11, *z* = 2.05, *p =* 0.041). No interaction was significant for depression severity, but the number of traumatic events showed a positive association with the sum scores (Table 1). Men reported fewer depressive symptoms than women (*ß* = -.23, SE = .10, *z* = -2.39, *p =* 0.017), and the experience of new traumatic events since baseline predicted higher sum scores (*ß* = .23, SE = .08, *z* = 2.98, *p =* 0.003).

**Table 1:** GLMMs summary for PTSD and depression symptom severity (clinical outcomes). GLMMs were used to test the predictors of NETfacts (no direct vs. direct involvement), time (baseline vs. post follow up), trauma (number of traumatic events), and their interaction on PTSD and depression symptom severity. Marginal and conditional R^2^ fit, and dispersion test estimates are shown for each outcome. Standardized model estimates, standard errors, standardized confidence intervals, *z* and *p* values are shown for the predictor terms. Random terms are represented as variance, standard deviation and sample size. Note that only the count model part of the zero-inflated GLMMs is shown here. Full GLMMs summaries including *post hoc* Tukey test for PSS-I are shown in S3 and S4 Tables.

**PTSD symptom severity (PSS-I)**					
**[Zero-inflated truncated Poisson GLMM; R**^**2**^ **= .20/.99; dispersion = .77, *p =* 0.192]**					
*Predictor terms*	***ß***	**SE**	***CI***	***z***	***p***
Intercept	2.19	.21	[1.78: 2.59]	10.60	**< 0.001**
NETfacts	-.08	.17	[-.41: .25]	-.45	0.65
Time	.00	.12	[-.23: .24]	.02	0.98
Trauma	.06	.18	[-.29: .40]	.33	0.75
*Interaction term*					
NETfacts: time: trauma	-.43	.13	[-.67: -.18]	-3.43	**0.001**
*Random terms*	**variance**	**SD**	**n**		
Participant	.39	.63	200		
Interviewer	.14	.37	17		
**Depression symptom severity (PHQ-9)**					
**[Zero-inflated truncated Poisson GLMM; R**^**2**^ **= .30/.96; dispersion = .97, *p =* 0.880]**					
*Predictor terms*	***ß***	**SE**	***CI***	***z***	***p***
Intercept	1.71	.14	[1.44: 1.98]	12.39	**< 0.001**
NETfacts	.19	.11	[-.02: .40]	1.74	0.08
Time	.03	.04	[-.05: .11]	.73	0.47
Trauma	.16	.04	[.07: .24]	3.61	**< 0.001**
*Interaction terms*					
NETfacts: time	-	-	-	-	ns
NETfacts: time: trauma	-	-	-	-	ns
*Random terms*	**variance**	**SD**	**n**		
Participant	.16	.40	199		
Interviewer	.07	.26	17		

ns: non-significant interaction effect.

#### Social outcomes

Table 2 shows GLMM summaries for SoRS, IRMA and SAQ. For SoRS, interactions were not significant, but the effect of time was associated with a decrease in sum scores as were more years of education (*ß* = -.07, SE = .03, *z* = -2.76, *p =* 0.006). A significant three-term interaction effect was found for IRMA: participants with a high number of traumatic events who were directly involved in NETfacts showed decreased sum scores at post follow up. An opposite trend was shown for no direct involvement. Further, male sex (*ß* = .08, SE = .04, *z* = 2.24, *p =* 0.025) and less years of education (*ß* = -.05, SE = .02, *z* = -2.72, *p =* 0.007) explained higher sum scores of IRMA. For SAQ, no interaction effect was found but significant effects of time and number of traumatic events. More years of education were negatively related to SAQ sum scores (*ß* = -.16, SE = .06, *z* = -2.80, *p =* 0.005).

**Table 2:** GLMMs summary of the final models for disbeliefs in social reconstruction with ex-combatants, rape myths acceptance and social acknowledgement as trauma survivor (social outcomes). GLMMs were used to test the predictors of NETfacts (no direct vs. direct involvement), time (baseline vs. post follow up), trauma (number of traumatic events), and their interaction on sum scores for SoRS, IRMA and SAQ. Marginal and conditional R^2^ fit, and dispersion test estimates are shown for each outcome. Standardized model estimates, standard errors, standardized confidence intervals, *z* and *p* values are shown for the predictor terms. Random terms are represented as variance, standard deviation and sample size. Note that only the count model part of the zero-inflated GLMMs is shown here. Full GLMMs summaries including figures (and *post hoc* Tukey test for IRMA) are shown in S5–S7 Tables and S1–S3 Figs.

**Disbeliefs in social reconstruction with ex-combatants (SoRS)**					
**[Poisson GLMM; R**^**2**^ **= .15/.73; dispersion = 1.0, *p =* 0.504]**					
*Predictor terms*	***ß***	**SE**	***CI***	***z***	***p***
Intercept	3.37	.07	[3.23: 3.51]	47.13	**< 0.001**
NETfacts	.06	.06	[-.05: .17]	1.09	0.27
Time	-.19	.02	[-.23: -.15]	-9.57	**< 0.001**
Trauma	.01	.02	[-.04: .06]	.51	0.61
*Interaction terms*					
NETfacts: time	-	-	-	-	ns
NETfacts: time: trauma	-	-	-	-	ns
*Random terms*	**variance**	**SD**	**n**		
Participant	.06	.25	200		
Interviewer	.02	.13	17		
**Rape myths acceptance (IRMA)**					
**[Poisson GLMM; R**^**2**^ **= .07/.6; dispersion = 1.1, *p =* 0.112]**					
*Predictor terms*	***ß***	**SE**	***CI***	***z***	***p***
Intercept	3.41	.06	[3.29: 3.53]	56.59	**< 0.001**
NETfacts	.01	.05	[-.08: .10]	.14	0.89
Time	.01	.04	[-.09: .09]	.18	0.85
Trauma	.03	.05	[-.07: .13]	.61	0.54
*Interaction terms*					
NETfacts: time: trauma	-.15	.05	[-.25: -.04]	-2.79	**0.005**
*Random terms*	**variance**	**SD**	**n**		
Participant	.02	.15	200		
Interviewer	.02	.14	17		
**Perceived lack of social acknowledgement as trauma survivor (SAQ)**					
**[Zero-inflated truncated Poisson GLMM; R**^**2**^ **= .23/.9; dispersion = .96, *p =* 0.776]**					
*Predictor terms*	***ß***	**SE**	***CI***	***z***	***p***
Intercept	1.39	.16	[1.08: 1.70]	8.88	**< 0.001**
NETfacts	.07	.12	[-.17: .31]	.58	0.56
Time	.13	.06	[.01: .26]	2.11	**0.035**
Trauma	.19	.05	[.09: .29]	3.70	**< 0.001**
*Interaction terms*					
NETfacts: time	-	-	-	-	ns
NETfacts: time: trauma	-	-	-	-	ns
*Random terms*	**variance**	**SD**	**n**		
Participant	.13	.37	189		
Interviewer	.07	.27	17		

ns: non-significant interaction effect.

Paired sample t-tests for ABSV showed an increased in affirmations of the item “willingness to support a family members affected by sexual violence” in those directly involved in NETfacts (*t*(159) = 3.43, *p* = 0.001, N = 200). No time difference was found in the other ABSV items dependent on NETfacts involvement (*p* > .05).

## Discussion

This study demonstrated the feasibility of a combined individual and community-based intervention that aims to decelerate the cycles of violence by breaking the secrecy surrounding trauma at both the individual and collective level. With it, a stepped care model was realised including (1) a screen-to-treat approach to refer trauma survivors with clinically relevant symptoms to treatment in the local health system (NET) and (2) a community intervention to mitigate the various effects of violence and their subclinical mental sequelae at the community level (NETfacts). Individuals who had experienced multiple traumatic events in their own past were most willing to engage in one of the offered activities (NETfacts community sessions including composite narratives, singular exposure session, NET after referral). Immediate beneficial effects were found for PTSD and rejection of rape myths among those directly involved in NETfacts who presented with multiple traumatic events in their past. Caveats to reconstruct social ties with ex-combatants were decreased at post follow up independent from NETfacts involvement. Those directly involved in NETfacts were more often willing to take care of a family member affected by sexual violence. No change was found in depression and an increase shown in the perceived lack of social acknowledgement as trauma survivor, independent from NETfacts.

### Feasibility of NETfacts

Every community bases its identity in parts on shared narratives. When traumatic events become more frequent, more individuals will suffer from PTSD and with it, a conspiracy of silence will prevail. The failure to connect threatening experiences to the relevant context and the resulting avoidance make it difficult to disclose, and social emotions like shame or guilt further inhibit reporting. Collective avoidance will magnify stigma and facilitate the perpetrator to conceal their crimes. As a result, atrocities and related suffering are not appropriately represented in the community’s shared account about the past. Therefore, it is in the interest of survivors to complete the shared knowledge by inclusion of the facts about the trauma. In general, survivors were open to provide an anonymized version of their NET testimonies for NETfacts. Despite their trauma-related difficulty in narrating and tendency to avoid trauma material, many feel strong desire and compulsion to share the distressing experience with others [101] and inform them about the violation of *shared* values that has occurred through the violent act. Accordingly, we found strong support for, and willingness to participate in, our activities. Individuals with respected local authority were highly committed to contribute with outreach activities and provide security updates. The various activities (diagnostic interviews, community sessions, singular exposure sessions, NET after referral) were well-attended. Shared composite narratives were being heard and discussed in a constructive way. Extreme psychological reactions were not observed during or after NETfacts sessions neither reported to the clinical professionals present in each session, suggesting clinical safety of the intervention. Altogether, this demonstrates that NETfacts is a feasible intervention. Moreover, it is also an effective tool to increase knowledge about available mental health services which increases an otherwise limited access of trauma survivors to treatment [31].

### Effects of NETfacts

Those directly involved in NETfacts who presented with a high trauma load showed reduced PTSD severity at post follow up. This may be a result of the exposure treatment (singular exposure session or NET), in line with prior research indicating that a minimal number of NET sessions (one to five) can reverse the building block effect and improve mental health problems [65, 71, 102]. NETfacts is unique in the active involvement of survivors’ broader social environment in recovery, and the encouraged discussion of collectively relevant traumatic events. Beyond the direct effects of exposure treatment, NETfacts thus may contribute a sense of being heard and understood by breaking the toxic secrecy surrounding traumatic material and increase feelings of social pain and stigma among survivors–conditions that have been associated with PTSD severity [103, 104]. Among the community, it may further induce others’ ability to anticipate survivors’ needs and elevate attempts for inclusion.

Individuals who were directly involved in NETfacts and presented with a low trauma load showed a statistically significant but clinically irrelevant increase of PTSD symptom severity values from baseline to post follow up (cf. Fig 2 and S3B Table). While it may be possible that learning about the psychic content of trauma which one’s family, friends or other members of the community have experienced accounts for this increased score [20], education about PTSD symptoms as such may also have led to increased reporting.

No such changes were found for depression. This was not the target of NETfacts. It is unlikely that an intervention which informs and stimulates discussion about traumatic experiences will immediately alter depression. However, PTSD and depression symptom networks are strongly interconnected [105, 106] and improvements in trauma-related symptoms usually come with (delayed) decreases in comorbid depression, as shown after a full NET treatment [62]. These changes would not be reflected in this feasibility study as no long-term follow-up was undertaken, given the dynamic changes in war zones.

Disbeliefs in social reconstruction with ex-combatants were decreased at post follow up. This is in line with discussions in community sessions in which participants agreed to consider the reintegration of ex-combatants, if they proved to be willing to adopt to non-violent civilian life and did not harm family and friends. This is an important finding as community stigma has shown to be associated with both ex-combatants’ mental health problems and post-combat aggression [58]. Most rehabilitation and reintegration programs have neglected civilians’ perspectives and concerns and often drew an almost unidimensional image of ex-combatants as victims while downplaying the impact of perpetrated acts on the individual and society, especially in the rehabilitation of child soldiers [107]. Whilst civilians often held ex-combatants less responsible for past perpetration after participating in these programs, reintegration obstacles often remained [108]. Disseminating and discussing NET testimonies of ex-combatants that include traumatizing experiences of severe perpetration does justice (‘truth’) to the complexity of trauma and allows the experiences of all community members to enter the collective memory, as well as allowing realistic discussion of rehabilitation options.

Rape myths acceptance showed a reversed trend at post follow up among those directly involved in NETfacts, whereby rejection of rape myths was stronger the higher the individual’s own past trauma exposure. Participants directly involved in NETfacts also indicated increased willingness to care for victimized family members. An important component of NETfacts is the provision of a forum to express emotional responses to others’ traumatic experiences. Discussions following the dissemination of testimonies may be essential for overcoming collective avoidance and encouraging prosocial behavior towards the traumatized. While holding on to rape myths serves the purpose of coping with the anxiety of the possibility of future victimization [36, 39, 109], the expression of emotional responses to survivors’ trauma presents an alternative coping mechanism and thus may reduce fear and victim blaming [72]. Reducing rape myths is important as stigma not only aggravates survivors’ trauma-related mental health problems [103, 104] but can also increase shame among the victims who often internalize the myths [110, 111], ultimately inducing hesitation to seek professional help from doctors, therapists or local authorities.

The perceived lack of social acknowledgement as a trauma survivor was increased post follow up. It is plausible that hearing stories about others’ trauma and stigmatization–information which is often socially tabooed, kept secret and unknown by others–increases the awareness that survivors lack social recognition in the community. Elliot and Devine [112] argued that psychological discomfort roots in cognitive dissonance and is the premise for attitude change. In the long-term, an increase in the perceived lack of social acknowledgement may, therefore, result in changes of negative attitudes towards marginalized survivor groups and a collective adoption of a more supportive stance towards trauma survivors would then, ultimately, increase feelings of social recognition within the community. Stigma, perceived lack of social acknowledgement and social support are all associated with PTSD symptoms [88–90, 103, 113]. Addressing these factors is therefore important to enable recovery from the traumatic experience and facilitate reengagement with everyday life, especially for survivors with elevated but not yet clinically relevant symptoms who do not require therapeutic treatment. In this case, despite the initial minor aggravations in SAQ, NETfacts could be preventative for survivors requiring professional care. Further research is required to explore the longitudinal development.

Particularly for social outcomes (SoRS, SAQ), changes over time were observed independent from NETfacts involvement. This may represent a spill-over effect from participants in addition to other factors associated with the project administration (e.g., observing people coming to the village to offer support). Xie, Sreenivasan [114] used computational models to study the spread of opinions across social networks and found that if a committed minority that continuously represents its opinion in public reaches a threshold of only 10%, their belief rapidly spills over and reverses opinions in the entire social network. However, Efferson, Vogt [115] highlighted that a strong heterogeneity in a community’s opinions and a high degree to which this stance contributes to the collective identity (esp. in the case of culturally accepted violence) can substantially slow down the spill-over effects. To achieve the best possible results, the authors advise to include a representative sample in interventions. In this study, a representative sample of the community was selected and invited all to participate in NETfacts. Though results for SoRS and SAQ independent from NETfacts may indicate a spill-over effect, this investigation should be a research target of future studies.

### Limitations

The main objective of this study was to test the feasibility of NETfacts and to explore effective strategies to moderate community sessions, in preparation for a longitudinal study including trained local moderators. The choice of an international moderator (British licenced clinical psychologist) as co-facilitator together with a Congolese moderator was unavoidable in this early stage of development and piloting of the intervention to test the moderation strategies and ensure clinical safety. Therefore, as originally planned, local moderators have now been trained in the approach and our ongoing research includes solely local moderators. This study was conducted in just one village. Assignment to the two groups direct vs no direct involvement was not random but self-determined. Spill-over effects from individuals directly involved in the intervention on other community members are possible, and desirable. This remains an interesting area of future investigation.

## Conclusions

Collective avoidance of traumatic material accelerates the cycles of violence in post conflict regions such as the Eastern DRC by protecting the perpetrators and marginalising the victims. The NETfacts health system effectively combines individual evidence based, narrative trauma therapy (NET) with a community based intervention (NETfacts) to share and initiate processing of avoided, collectively relevant facts about trauma. Though areas of conflict and organized violence may initially appear most appropriate target sites, iterating human right violations pervade populations all around the globe, can create a societal split between traumatized minorities and their social realm and promote further unrest. Giving voice to those who have been victimized and suppressed due to trauma-related, cultural, ethnical, social or sexual characteristics holds the potential for perspective adoption, social reapproach and, ultimately, reconciliation.

## Supporting information

S1 Fig Significant effect of time on SoRS. (DOCX)

S2 Fig Significant interaction effect of the number of traumatic events among participants with no direct vs direct involvement in NETfacts from baseline to post follow up (time marked in color) on IRMA. (DOCX)

S3 Fig Significant effect of time on SAQ. (DOCX)

S1 Table Pre-survey statistics on number of households, and total, representative and final population size (assessed at baseline and post follow up). (DOCX)

S2 Table Demographic information for participants of group discussion during the development of the NETfacts community intervention. (DOCX)

S3 Table A. GLMMs summary of the final model for PSS-I. B. Post hoc Tukey tests for PSS-I.
(DOCX)

S4 Table GLMMs summary of the final model for PHQ-9. (DOCX)

S5 Table GLMMs summary of the final model for SoRS. (DOCX)

S6 Table A. GLMMs summary of the final model for IRMA. B. Post hoc Tukey tests for IRMA.
(DOCX)

S7 Table GLMMs summary of the final model for SAQ. (DOCX)
