# Supplementary figures and images for: Breaking the cycles of violence with narrative exposure: Development and feasibility of NETfacts, a community-based intervention for populations living under continuous threat
Source: PLoS One. 2022 Dec 19;17(12):e0275421. doi: 10.1371/journal.pone.0275421 (PMC9762574; doi:10.1371/journal.pone.0275421)

**S1 Figure. Significant effect of time on SoRS.**
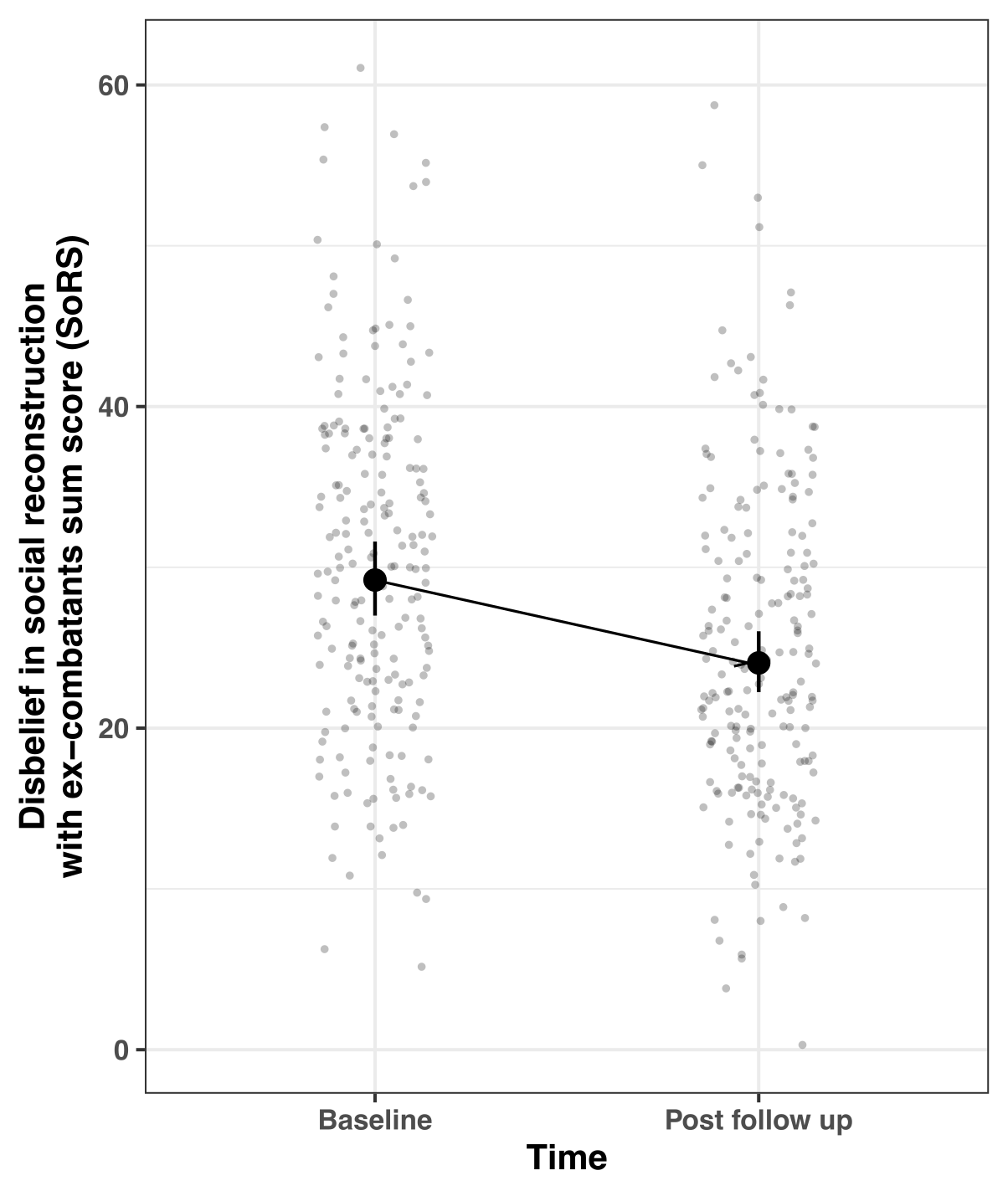

Supplement: S1 Fig — (DOCX) [file pone.0275421.s001.docx]

**S3 Figure. Significant effect of time on SAQ.**


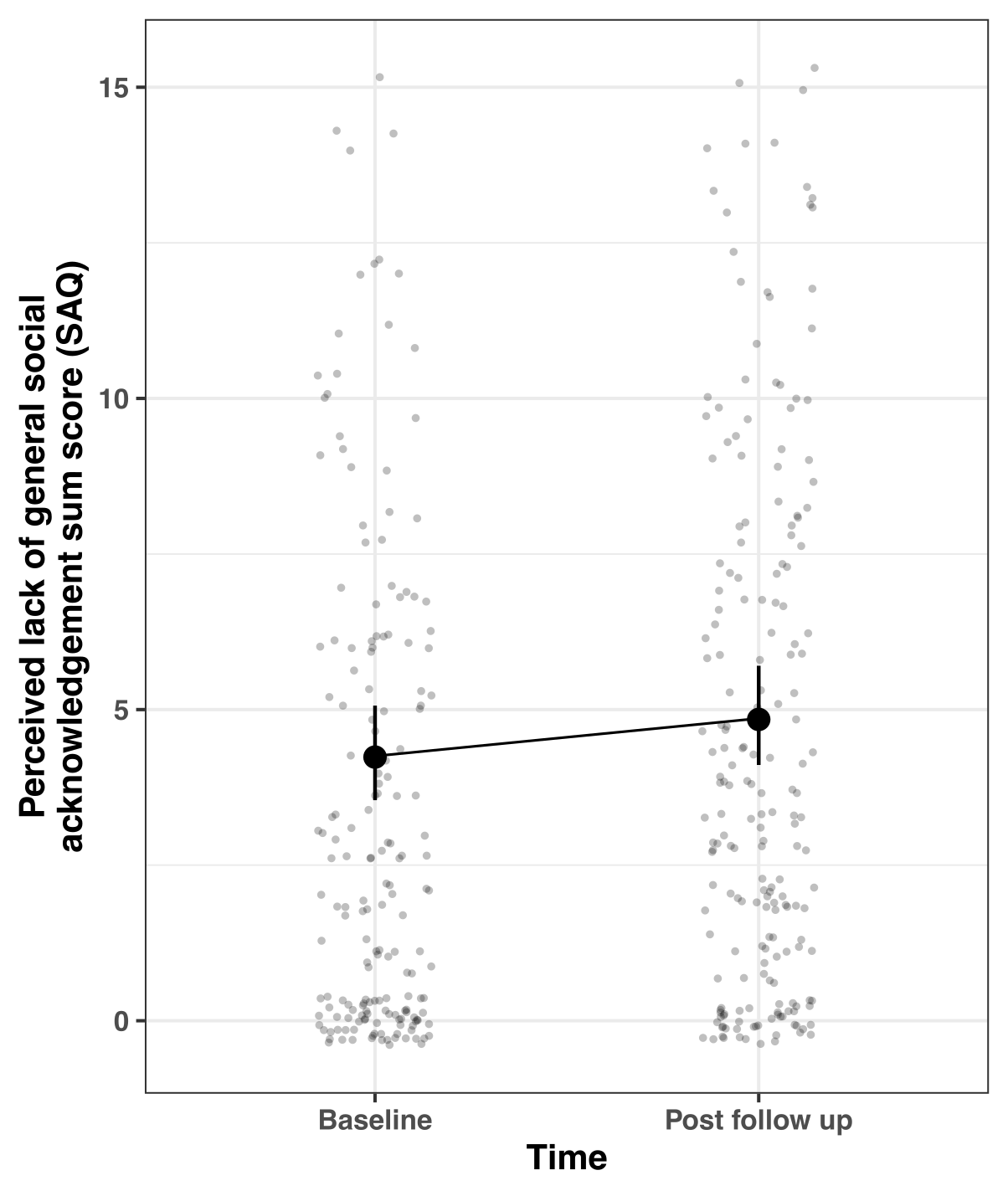

Supplement: S3 Fig — (DOCX) [file pone.0275421.s003.docx]
