# Supplementary material for: Breaking the cycles of violence with narrative exposure: Development and feasibility of NETfacts, a community-based intervention for populations living under continuous threat
Source: PLoS One. 2022 Dec 19;17(12):e0275421. doi: 10.1371/journal.pone.0275421 (PMC9762574; doi:10.1371/journal.pone.0275421)
Supplement: S2 Fig — (DOCX) [file pone.0275421.s002.docx]

**S2 Figure. Significant interaction effect of the number of traumatic events among participants with no direct vs direct involvement in NETfacts from baseline to post follow up (time marked in color) on IRMA.**

**
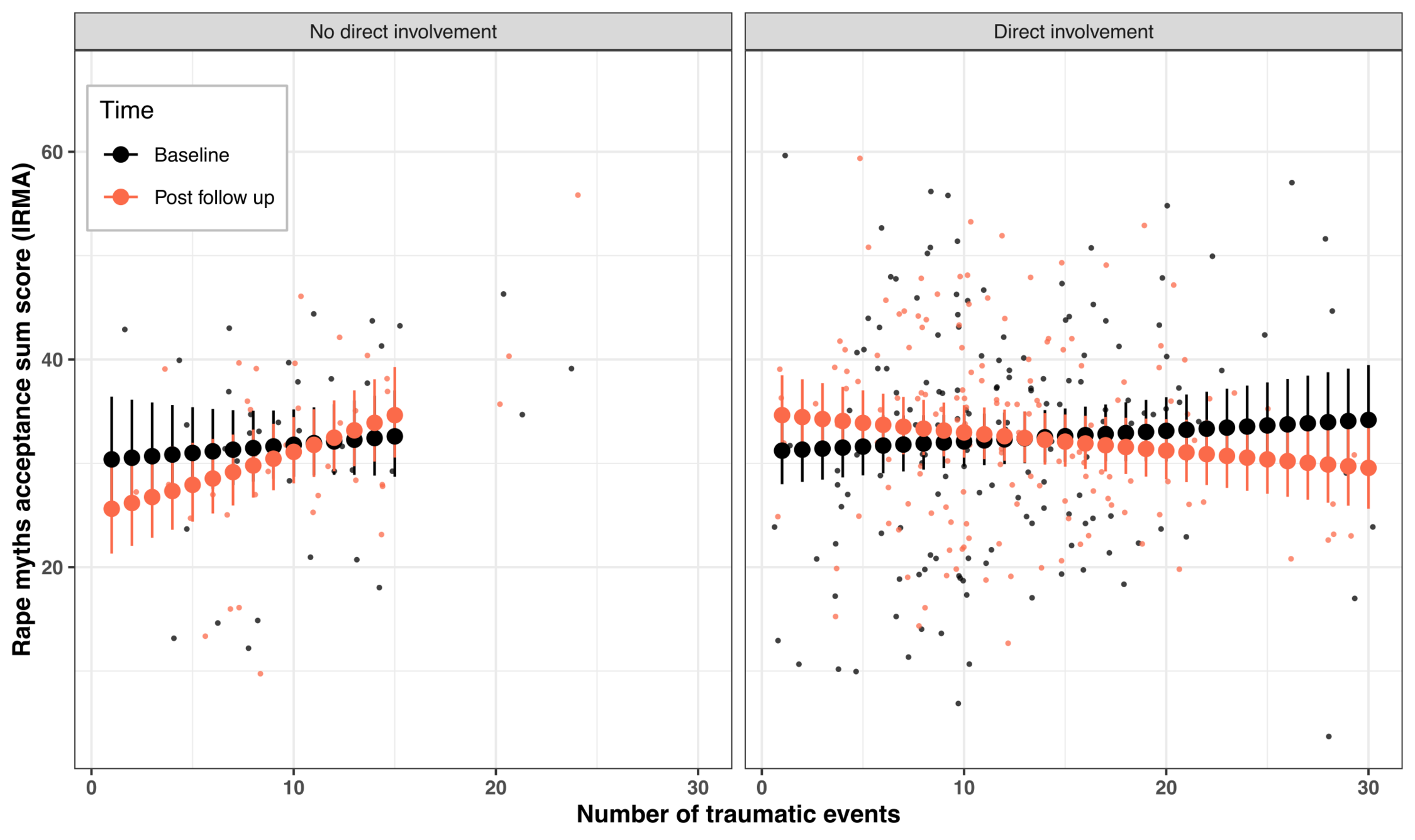
**
