# Supplementary material for: Breaking the cycles of violence with narrative exposure: Development and feasibility of NETfacts, a community-based intervention for populations living under continuous threat
Source: PLoS One. 2022 Dec 19;17(12):e0275421. doi: 10.1371/journal.pone.0275421 (PMC9762574; doi:10.1371/journal.pone.0275421)
Supplement: S1 Table — (DOCX) [file pone.0275421.s004.docx]

# **Supporting information**

S1 Table. Pre-survey statistics on number of households, and total, representative and final population size (assessed at baseline and post follow up).

|  | **Total** | **Men** | **Women** |
| --- | --- | --- | --- |
| Number of households | 205 |  |  |
| Total population size |  |  |  |
| Total | 497 | 240 | 257 |
| Age 16 to 36 | 287 | 138 | 149 |
| Age 37 to 57 | 125 | 49 | 76 |
| Over age 57 | 85 | 53 | 32 |
| Representative population size |  |  |  |
| Total | 216 | 106 | 110 |
| Age 16 to 36 | 115 | 56 | 59 |
| Age 37 to 57 | 59 | 24 | 35 |
| Over age 57 | 42 | 26 | 16 |
| Final population size |  |  |  |
| Total | 200 | 93 | 107 |
| Age 16 to 36 | 105 | 50 | 55 |
| Age 37 to 57 | 55 | 18 | 37 |
| Over age 57 | 40 | 25 | 15 |
