# Supplementary material for: Breaking the cycles of violence with narrative exposure: Development and feasibility of NETfacts, a community-based intervention for populations living under continuous threat
Source: PLoS One. 2022 Dec 19;17(12):e0275421. doi: 10.1371/journal.pone.0275421 (PMC9762574; doi:10.1371/journal.pone.0275421)
Supplement: S2 Table — (DOCX) [file pone.0275421.s005.docx]

**S2 Table. Demographic information for participants of group discussion during the development of the NETfacts community intervention (N = 66)** ^1^**.** Values are presented as medians and ranges for age, years of education and number of children, and as frequencies and percentages for sex and relationship status.

|  | **G1**  **(n = 6)** | **G2**  **(n = 3)** | **G3**  **(n = 5)** | **G4**  **(n = 3)** | **G5**  **(n = 8)** | **G6**  **(n = 9)** | **G7**  **(n = 8)** | **G8**  **(n = 8)** | **G9**  **(n = 8)** | **G10**  **(n = 8)** |
| --- | --- | --- | --- | --- | --- | --- | --- | --- | --- | --- |
| Females | 3 (50) | 3 (100) | 5 (100) | 3 (100) | 8 (100) | 0 (0) | 0 (0) | 3 (38) | 0 (0) | 4 (50) |
| Age | 31 (27-45) | 38 (26-48) | 47 (27-67) | 42 (28-50) | 19 (17-27) | 37 (26-52) | 34 (20-41) | 39 (28-59) | 17 (16-19) | 44 (27-59) |
| Years of education | 16 (12-20) | 12 (12-12) | 9 (4-12) | 7 (0-10) | 10 (7-12) | 8 (0-12) | 9 (6-12) | 12 (12-12) | 8 (4-11) | 10 (4-12) |
| Relationship status |  |  |  |  |  |  |  |  |  |  |
| Single | 6 (100) | 1 (33) | 1 (20) | 2 (67) | 7 (88) | 1 (11) | 1 (13) | 0 (0) | 6 (86) | 0 (0) |
| Married/with partner | 0 (0) | 2 (67) | 2 (40) | 1 (33) | 1 (12) | 8 (89) | 7 (87) | 7 (88) | 1 (14) | 6 (75) |
| Number of children | 1 (0-2) | 4 (2-6) | 7 (3-14) | 5 (4-6) | 0 (0-1) | 5 (0-9) | 3 (0-6) | 3 (0-8) | 0 (0-0) | 6 (1-15) |

*Note.* G1: NET counsellors, G2: coordinators of non-governmental organizations (NGO) working with rape survivors, G3: focal points of NGOs working with rape survivors, G4: female rape survivors, G5: female ex-combatants resettled in rural community, G6: community sensitizers against social rejection of rape survivors, G7: male ex-combatants living in Goma, not resettled yet in rural community, G8: focal points of NGOs working with ex-combatants and FORNET counsellor, G9: male ex-combatants resettled in rural community, G10: community members without history of rape or combatant life

^1^ Procedure. Group discussions were conducted between February and April 2018 in Goma, EDRC. Maximum variation purposive sampling was used to select participants. The number of ten group discussions seemed sufficient based on prior propositions that usually 80% of all themes, especially the most prevalent, are revealed in two to three distinct group discussions and 90% in three to six [1]. Participants were eligible if they were at least 16 years old. One international and seven national NGOs on-site facilitated the recruitment process. Discussions took place at the venue of the NGOs or in our local office. Participants received light refreshment and financial compensation (8.000 CFC, equivalent to 5$). No participant dropped out during the discussions. Discussions were moderated by two facilitators following a semi-structured manual, lasted two to three hours, were audio-recorded, translated from the local dialect Kiswahili into English, anonymised, transcribed and stored together with notes made during and shortly after the discussions. The first two group discussions were moderated by the first author and the remaining discussions by a native Congolese with the help of a colleague to diminish the likelihood of impaired conversational dynamics due to perceived (intercultural) discrepancy [2].

# **References**

**1.** Guest G, Namey E, McKenna K. How many focus groups are enough? Building an evidence base for nonprobability sample sizes. Field methods. 2017;29(1):3-22.

**2.** Tausch AP, Menold N. Methodological aspects of focus groups in health research: results of qualitative interviews with focus group moderators. Global Qualitative Nursing Research. 2016;3:2333393616630466.
