# Supplementary material for: Breaking the cycles of violence with narrative exposure: Development and feasibility of NETfacts, a community-based intervention for populations living under continuous threat
Source: PLoS One. 2022 Dec 19;17(12):e0275421. doi: 10.1371/journal.pone.0275421 (PMC9762574; doi:10.1371/journal.pone.0275421)
Supplement: S3 Table — A. GLMMs summary of the final model for PSS-I. B. Post hoc Tukey tests for PSS-I. (DOCX) [file pone.0275421.s006.docx]

# **Supporting information**

**S3 Table A. GLMMs summary of the final model for PSS-I.**

| **PTSD symptom severity (PSS-I)**  **[Zero-inflated truncated Poisson GLMM; R^2^ = .20/.99; dispersion = .77, *p* = .192]** | | | | | |
| --- | --- | --- | --- | --- | --- |
| **Count Model** |  |  |  |  |  |
| *Predictor terms* | ***ß*** | **SE** | ***CI*** | ***z*** | ***p*** |
| intercept | 2.19 | .21 | [1.78: 2.59] | 10.60 | **< .001** |
| NETfacts | -.08 | .17 | [-.41: .25] | -.45 | .650 |
| time | .00 | .12 | [-.23: .24] | .02 | .983 |
| trauma | .06 | .18 | [-.29: .40] | .33 | .745 |
| *Covariates* |  |  |  |  |  |
| new trauma since baseline | .22 | .11 | [.01: .44] | 2.05 | **.041** |
| perpetration of violent acts | .04 | .07 | [-.09: .18] | .66 | .508 |
| male sex | -.25 | .14 | [-.52: .02] | -1.82 | .069 |
| age | -.03 | .06 | [-.15: .09] | -.42 | .676 |
| years of education | -.05 | .07 | [-.18: .08] | -.77 | .444 |
| *Interaction terms* |  |  |  |  |  |
| NETfacts : time : trauma | -.43 | .13 | [-.67: -.18] | -3.43 | **.001** |
| NETfacts : time | .10 | .13 | [-.15: .36] | .80 | .423 |
| **Zero-Inflated Model** |  |  |  |  |  |
| *Predictor terms* |  |  |  |  |  |
| intercept | -1.12 | .61 | [-2.31: .08] | -1.83 | .067 |
| NETfacts | -.38 | .59 | [-1.54: .78] | -.64 | .522 |
| time | .61 | .64 | [-.65: 1.87] | .94 | .345 |
| trauma | -1.21 | .66 | [-2.51: .10] | -1.81 | .070 |
| *Covariates* |  |  |  |  |  |
| new trauma since baseline | -.74 | .29 | [-1.31: -.17] | -2.55 | **.011** |
| perpetration of violent acts | -.26 | .19 | [-.64: .12] | -1.32 | .187 |
| male sex | .01 | .33 | [-.64: .65] | .02 | .987 |
| age | .09 | .09 | [-.20: .37] | .60 | .549 |
| years of education | .27 | .16 | [-.06: .59] | 1.61 | .106 |
| *Interaction terms* |  |  |  |  |  |
| NETfacts : time : trauma | .26 | .90 | [-1.51: 2.03] | .29 | .775 |
| NETfacts : time | -.19 | .72 | [-1.61: 1.22] | -.27 | .788 |
| *Random terms* | **variance** | **SD** | **n** |  |  |
| participant | .39 | .63 | 200 |  |  |
| interviewer | .14 | .37 | 17 |  |  |

**S3 Table B. *Post hoc* Tukey tests for PSS-I.** Within- and between-group differences in PSS-I at baseline and post follow up are indicated for no direct vs direct involvement (N = 200). Tests calculated based on model predictions are marked with a number sign.

|  | | ***n_nodirect_*** | ***n_direct_*** | ***estimate*** | ***SE*** | ***t*** | ***p*** |
| --- | --- | --- | --- | --- | --- | --- | --- |
| # Number of traumatic events = 1 | | 0 | 4 |  |  |  |  |
| *WG* | No direct involvement T0 vs T1 |  |  | 2.22 | 1.94 | 1.15 | .660 |
|  | Direct involvement T0 vs T1 |  |  | -2.90 | .74 | -3.92 | <.001 |
| *BG* | T0 no direct vs direct involvement |  |  | 4.14 | 2.66 | 1.56 | .406 |
|  | T1 no direct vs direct involvement |  |  | -.98 | 2.00 | -.49 | .961 |
| Number of traumatic events = 2 | | 1 | 1 |  |  |  |  |
| *WG* | No direct involvement T0 vs T1 |  |  | 2.07 | 1.82 | 1.13 | .669 |
|  | Direct involvement T0 vs T1 |  |  | -2.81 | .71 | -3.97 | <.001 |
| *BG* | T0 no direct vs direct involvement |  |  | 3.94 | 2.49 | 1.58 | .389 |
|  | T1 no direct vs direct involvement |  |  | -.94 | 1.92 | -.49 | .962 |
| # Number of traumatic events = 3 | | 0 | 1 |  |  |  |  |
| *WG* | No direct involvement T0 vs T1 |  |  | 1.90 | 1.71 | 1.11 | .683 |
|  | Direct involvement T0 vs T1 |  |  | -2.71 | .68 | -4.01 | <.001 |
| *BG* | T0 no direct vs direct involvement |  |  | 3.71 | 2.31 | 1.61 | .376 |
|  | T1 no direct vs direct involvement |  |  | -.89 | 1.84 | -.48 | .963 |
| Number of traumatic events = 4 | | 2 | 8 |  |  |  |  |
| *WG* | No direct involvement T0 vs T1 |  |  | 1.72 | 1.60 | 1.08 | .704 |
|  | Direct involvement T0 vs T1 |  |  | -2.59 | .65 | -4.01 | <.001 |
| *BG* | T0 no direct vs direct involvement |  |  | 3.47 | 2.14 | 1.62 | .368 |
|  | T1 no direct vs direct involvement |  |  | -.84 | 1.76 | -.48 | .964 |
| Number of traumatic events = 5 | | 2 | 5 |  |  |  |  |
| *WG* | No direct involvement T0 vs T1 |  |  | 1.53 | 1.49 | 1.03 | .734 |
|  | Direct involvement T0 vs T1 |  |  | -2.45 | .62 | -3.99 | <.001 |
| *BG* | T0 no direct vs direct involvement |  |  | 3.20 | 1.98 | 1.62 | .369 |
|  | T1 no direct vs direct involvement |  |  | -.78 | 1.67 | -.47 | .966 |
| Number of traumatic events = 6 | | 2 | 7 |  |  |  |  |
| *WG* | No direct involvement T0 vs T1 |  |  | 1.34 | 1.39 | .96 | .773 |
|  | Direct involvement T0 vs T1 |  |  | -2.30 | .59 | -3.92 | <.001 |
| *BG* | T0 no direct vs direct involvement |  |  | 2.91 | 1.83 | 1.59 | .384 |
|  | T1 no direct vs direct involvement |  |  | -.72 | 1.59 | -.45 | .969 |
| Number of traumatic events = 7 | | 6 | 8 |  |  |  |  |
| *WG* | No direct involvement T0 vs T1 |  |  | 1.13 | 1.30 | .87 | .821 |
|  | Direct involvement T0 vs T1 |  |  | -2.12 | .56 | -3.81 | <.001 |
|  | T0 no direct vs direct involvement |  |  | 2.60 | 1.69 | 1.53 | .419 |
|  | T1 no direct vs direct involvement |  |  | -.65 | 1.52 | -.43 | .973 |

|  | | ***n_nodirect_*** | ***n_direct_*** | ***estimate*** | ***SE*** | ***t*** | ***p*** |
| --- | --- | --- | --- | --- | --- | --- | --- |
| Number of traumatic events = 8 | | 6 | 13 |  |  |  |  |
| *WG* | No direct involvement T0 vs T1 |  |  | .91 | 1.22 | .75 | .878 |
|  | Direct involvement T0 vs T1 |  |  | -1.92 | .53 | -3.63 | .002 |
| *BG* | T0 no direct vs direct involvement |  |  | 2.25 | 1.58 | 1.43 | .485 |
|  | T1 no direct vs direct involvement |  |  | -.58 | 1.45 | -.40 | .978 |
| Number of traumatic events = 9 | | 1 | 9 |  |  |  |  |
| *WG* | No direct involvement T0 vs T1 |  |  | .68 | 1.15 | .59 | .934 |
|  | Direct involvement T0 vs T1 |  |  | -1.70 | .51 | -3.36 | .005 |
| *BG* | T0 no direct vs direct involvement |  |  | 1.88 | 1.50 | 1.25 | .593 |
|  | T1 no direct vs direct involvement |  |  | -.50 | 1.41 | -.36 | .985 |
| Number of traumatic events = 10 | | 4 | 20 |  |  |  |  |
| *WG* | No direct involvement T0 vs T1 |  |  | .44 | 1.10 | .40 | .978 |
|  | Direct involvement T0 vs T1 |  |  | -1.45 | .48 | -3.00 | .016 |
| *BG* | T0 no direct vs direct involvement |  |  | 1.47 | 1.45 | 1.01 | .741 |
|  | T1 no direct vs direct involvement |  |  | -.42 | 1.40 | -.30 | .991 |
| Number of traumatic events = 11 | | 2 | 6 |  |  |  |  |
| *WG* | No direct involvement T0 vs T1 |  |  | .19 | 1.07 | .18 | .998 |
|  | Direct involvement T0 vs T1 |  |  | -1.17 | .47 | -2.51 | .060 |
| *BG* | T0 no direct vs direct involvement |  |  | 1.03 | 1.45 | .71 | .892 |
|  | T1 no direct vs direct involvement |  |  | -.33 | 1.43 | -.23 | .996 |
| Number of traumatic events = 12 | | 3 | 12 |  |  |  |  |
| *WG* | No direct involvement T0 vs T1 |  |  | -.08 | 1.08 | -.07 | 1.00 |
|  | Direct involvement T0 vs T1 |  |  | -.86 | .46 | -1.89 | .234 |
| *BG* | T0 no direct vs direct involvement |  |  | .55 | 1.50 | .37 | .983 |
|  | T1 no direct vs direct involvement |  |  | -.23 | 1.51 | -.15 | .999 |
| Number of traumatic events = 13 | | 2 | 9 |  |  |  |  |
| *WG* | No direct involvement T0 vs T1 |  |  | -.36 | 1.11 | -.32 | .988 |
|  | Direct involvement T0 vs T1 |  |  | -.52 | .46 | -1.15 | .662 |
| *BG* | T0 no direct vs direct involvement |  |  | .04 | 1.59 | .02 | 1.00 |
|  | T1 no direct vs direct involvement |  |  | -.12 | 1.63 | -.08 | 1.00 |
| Number of traumatic events = 14 | | 4 | 6 |  |  |  |  |
| *WG* | No direct involvement T0 vs T1 |  |  | -.65 | 1.18 | -.56 | .945 |
|  | Direct involvement T0 vs T1 |  |  | -.14 | .47 | -.31 | .990 |
| *BG* | T0 no direct vs direct involvement |  |  | -.52 | 1.73 | -.30 | .990 |
|  | T1 no direct vs direct involvement |  |  | -.01 | 1.81 | -.01 | 1.00 |
| Number of traumatic events = 15 | | 2 | 8 |  |  |  |  |
| *WG* | No direct involvement T0 vs T1 |  |  | -.96 | 1.28 | -.75 | .875 |
|  | Direct involvement T0 vs T1 |  |  | .27 | .49 | .56 | .945 |
| *BG* | T0 no direct vs direct involvement |  |  | -1.13 | 1.90 | -.59 | .934 |
|  | T1 no direct vs direct involvement |  |  | .11 | 2.03 | .05 | 1.00 |

|  | | ***n_nodirect_*** | ***n_direct_*** | ***estimate*** | ***SE*** | ***t*** | ***p*** |
| --- | --- | --- | --- | --- | --- | --- | --- |
| # Number of traumatic events = 16 | | 0 | 9 |  |  |  |  |
| *WG* | No direct involvement T0 vs T1 |  |  | -1.29 | 1.42 | -.91 | .800 |
|  | Direct involvement T0 vs T1 |  |  | .73 | .53 | 1.37 | .518 |
| *BG* | T0 no direct vs direct involvement |  |  | -1.78 | 2.11 | -.84 | .833 |
|  | T1 no direct vs direct involvement |  |  | .24 | 2.30 | .10 | 1.00 |
| # Number of traumatic events = 17 | | 0 | 5 |  |  |  |  |
| *WG* | No direct involvement T0 vs T1 |  |  | -1.63 | 1.59 | -1.03 | .734 |
|  | Direct involvement T0 vs T1 |  |  | 1.24 | .60 | 2.07 | .164 |
| *BG* | T0 no direct vs direct involvement |  |  | -2.49 | 2.35 | -1.06 | .715 |
|  | T1 no direct vs direct involvement |  |  | .37 | 2.62 | .14 | .999 |
| # Number of traumatic events = 18 | | 0 | 4 |  |  |  |  |
| *WG* | No direct involvement T0 vs T1 |  |  | -1.98 | 1.79 | -1.11 | .684 |
|  | Direct involvement T0 vs T1 |  |  | 1.79 | .68 | 2.63 | .045 |
| *BG* | T0 no direct vs direct involvement |  |  | -3.26 | 2.62 | -1.24 | .600 |
|  | T1 no direct vs direct involvement |  |  | .52 | 2.97 | .18 | .998 |
| # Number of traumatic events = 19 | | 0 | 3 |  |  |  |  |
| *WG* | No direct involvement T0 vs T1 |  |  | -2.36 | 2.02 | -1.17 | .647 |
|  | Direct involvement T0 vs T1 |  |  | 2.40 | .79 | 3.03 | .014 |
| *BG* | T0 no direct vs direct involvement |  |  | -4.08 | 2.91 | -1.40 | .499 |
|  | T1 no direct vs direct involvement |  |  | .68 | 3.36 | .20 | .997 |
| Number of traumatic events = 20 | | 1 | 6 |  |  |  |  |
| *WG* | No direct involvement T0 vs T1 |  |  | -2.75 | 2.28 | -1.21 | .624 |
|  | Direct involvement T0 vs T1 |  |  | 3.06 | .92 | 3.32 | .006 |
| *BG* | T0 no direct vs direct involvement |  |  | -4.97 | 3.23 | -1.54 | .415 |
|  | T1 no direct vs direct involvement |  |  | .84 | 3.80 | .22 | .996 |
| Number of traumatic events = 21 | | 1 | 5 |  |  |  |  |
| *WG* | No direct involvement T0 vs T1 |  |  | -3.16 | 2.57 | -1.23 | .609 |
|  | Direct involvement T0 vs T1 |  |  | 3.79 | 1.08 | 3.50 | .003 |
| *BG* | T0 no direct vs direct involvement |  |  | -5.93 | 3.58 | -1.66 | .347 |
|  | T1 no direct vs direct involvement |  |  | 1.02 | 4.27 | .24 | .995 |
| # Number of traumatic events = 22 | | 0 | 2 |  |  |  |  |
| *WG* | No direct involvement T0 vs T1 |  |  | -3.59 | 2.90 | -1.24 | .602 |
|  | Direct involvement T0 vs T1 |  |  | 4.58 | 1.27 | 3.60 | .002 |
| *BG* | T0 no direct vs direct involvement |  |  | -6.97 | 3.95 | -1.77 | .292 |
|  | T1 no direct vs direct involvement |  |  | 1.21 | 4.78 | .25 | .994 |
| # Number of traumatic events = 23 | | 0 | 1 |  |  |  |  |
| *WG* | No direct involvement T0 vs T1 |  |  | -4.04 | 3.25 | -1.24 | .600 |
|  | Direct involvement T0 vs T1 |  |  | 5.44 | 1.49 | 3.65 | .002 |
| *BG* | T0 no direct vs direct involvement |  |  | -8.08 | 4.35 | -1.86 | .248 |
|  | T1 no direct vs direct involvement |  |  | 1.41 | 5.33 | .26 | .994 |

|  | | ***n_nodirect_*** | ***n_direct_*** | ***estimate*** | ***SE*** | ***t*** | ***p*** |
| --- | --- | --- | --- | --- | --- | --- | --- |
| # Number of traumatic events = 24 | | 1 | 0 |  |  |  |  |
| *WG* | No direct involvement T0 vs T1 |  |  | -4.51 | 3.65 | -1.24 | .603 |
|  | Direct involvement T0 vs T1 |  |  | 6.39 | 1.75 | 3.66 | .002 |
| *BG* | T0 no direct vs direct involvement |  |  | -9.28 | 4.78 | -1.94 | .213 |
|  | T1 no direct vs direct involvement |  |  | 1.62 | 5.93 | .27 | .993 |
| # Number of traumatic events = 25 | | 0 | 1 |  |  |  |  |
| *WG* | No direct involvement T0 vs T1 |  |  | -5.01 | 4.07 | -1.23 | .608 |
|  | Direct involvement T0 vs T1 |  |  | 7.41 | 2.04 | 3.64 | .002 |
| *BG* | T0 no direct vs direct involvement |  |  | -10.58 | 5.25 | -2.01 | .185 |
|  | T1 no direct vs direct involvement |  |  | 1.84 | 6.57 | .28 | .992 |
| # Number of traumatic events = 26 | | 0 | 1 |  |  |  |  |
| *WG* | No direct involvement T0 vs T1 |  |  | -5.53 | 4.54 | -1.22 | .616 |
|  | Direct involvement T0 vs T1 |  |  | 8.53 | 2.37 | 3.60 | .002 |
|  | T0 no direct vs direct involvement |  |  | -11.97 | 5.76 | -2.08 | .163 |
|  | T1 no direct vs direct involvement |  |  | 2.08 | 7.25 | .29 | .992 |
| # Number of traumatic events = 27 | | 0 | 3 |  |  |  |  |
| *WG* | No direct involvement T0 vs T1 |  |  | -6.07 | 5.05 | -1.20 | .626 |
|  | Direct involvement T0 vs T1 |  |  | 9.74 | 2.75 | 3.54 | .003 |
| *BG* | T0 no direct vs direct involvement |  |  | -13.47 | 6.32 | -2.13 | .145 |
|  | T1 no direct vs direct involvement |  |  | 2.34 | 7.98 | .29 | .991 |
| # Number of traumatic events = 28 | | 0 | 0 |  |  |  |  |
| *WG* | No direct involvement T0 vs T1 |  |  | -6.64 | 5.60 | -1.19 | .636 |
|  | Direct involvement T0 vs T1 |  |  | 11.05 | 3.18 | 3.47 | .003 |
| *BG* | T0 no direct vs direct involvement |  |  | -15.08 | 6.92 | -2.18 | .131 |
|  | T1 no direct vs direct involvement |  |  | 2.60 | 8.77 | .30 | .991 |
| # Number of traumatic events = 29 | | 0 | 2 |  |  |  |  |
| *WG* | No direct involvement T0 vs T1 |  |  | -7.23 | 6.19 | -1.17 | .648 |
|  | Direct involvement T0 vs T1 |  |  | 12.48 | 3.67 | 3.40 | .004 |
|  | T0 no direct vs direct involvement |  |  | -16.82 | 7.58 | -2.22 | .120 |
|  | T1 no direct vs direct involvement |  |  | 2.89 | 9.60 | .30 | .991 |
| # Number of traumatic events = 30 | | 0 | 1 |  |  |  |  |
| *WG* | No direct involvement T0 vs T1 |  |  | -7.85 | 6.84 | -1.15 | .660 |
|  | Direct involvement T0 vs T1 |  |  | 14.03 | 4.22 | 3.32 | .005 |
| *BG* | T0 no direct vs direct involvement |  |  | -18.69 | 8.30 | -2.25 | .112 |
|  | T1 no direct vs direct involvement |  |  | 3.19 | 10.50 | .30 | .990 |
