# Supplementary material for: Breaking the cycles of violence with narrative exposure: Development and feasibility of NETfacts, a community-based intervention for populations living under continuous threat
Source: PLoS One. 2022 Dec 19;17(12):e0275421. doi: 10.1371/journal.pone.0275421 (PMC9762574; doi:10.1371/journal.pone.0275421)
Supplement: S4 Table — (DOCX) [file pone.0275421.s007.docx]

# **Supporting information**

**S4 Table. GLMMs summary of the final model for PHQ-9.**

| **Depression symptom severity (PHQ-9)**  **[Zero-inflated truncated Poisson GLMM; R^2^ = .30/.96; dispersion = .97, *p* = .880]** | | | | | |
| --- | --- | --- | --- | --- | --- |
| **Count Model** |  |  |  |  |  |
| *Predictor terms* | ***ß*** | **SE** | ***CI*** | ***z*** | ***p*** |
| intercept | 1.71 | .14 | [1.44: 1.98] | 12.39 | **<.001** |
| NETfacts | .19 | .11 | [-.02: .40] | 1.74 | .081 |
| time | .03 | .04 | [-.05: .11] | .73 | .468 |
| trauma | .16 | .04 | [.07: .24] | 3.61 | **<.001** |
| *Covariates* |  |  |  |  |  |
| new trauma since baseline | .23 | .08 | [.08: .38] | 2.98 | **.003** |
| perpetration of violent acts | .03 | .05 | [-.06: .12] | .64 | .521 |
| male sex | -.23 | .10 | [-.42: -.04] | -2.39 | **.017** |
| age | .07 | .04 | [-.01: .15] | 1.71 | .088 |
| years of education | -.08 | .05 | [-.17: .02] | -1.53 | .125 |
| *Interaction terms* |  |  |  |  |  |
| NETfacts : time : trauma | - | - | - | - | ns |
| NETfacts : time | - | - | - | - | ns |
| **Zero-Inflated Model** |  |  |  |  |  |
| *Predictor terms* |  |  |  |  |  |
| intercept | -3.07 | .77 | [-4.58: -1.55] | -3.97 | **<.001** |
| NETfacts | .72 | .52 | [-.29: 1.74] | 1.40 | .162 |
| time | -.07 | .35 | [-.75: .61] | -.21 | .832 |
| trauma | -.68 | .28 | [-1.24: -.13] | -2.41 | **.016** |
| *Covariates* |  |  |  |  |  |
| new trauma since baseline | -.63 | .42 | [-1.46: .20] | -1.49 | .136 |
| perpetration of violent acts | -.35 | .29 | [-.92: .22] | -1.21 | .226 |
| male sex | .10 | .47 | [-.83: 1.03] | .22 | .828 |
| age | -.34 | .23 | [-.79: .11] | -1.48 | .138 |
| years of education | .55 | .25 | [.07: 1.03] | 2.24 | **.025** |
| *Interaction terms* |  |  |  |  |  |
| NETfacts : time : trauma | - | - | - | - | ns |
| NETfacts : time | - | - | - | - | ns |
| *Random terms* | **variance** | **SD** | **n** |  |  |
| participant | .16 | .40 | 199 |  |  |
| interviewer | .07 | .26 | 17 |  |  |
