# Supplementary material for: Breaking the cycles of violence with narrative exposure: Development and feasibility of NETfacts, a community-based intervention for populations living under continuous threat
Source: PLoS One. 2022 Dec 19;17(12):e0275421. doi: 10.1371/journal.pone.0275421 (PMC9762574; doi:10.1371/journal.pone.0275421)
Supplement: S5 Table — (DOCX) [file pone.0275421.s008.docx]

# **Supporting information**

**S5 Table. GLMMs summary of the final model for SoRS.**

| **Disbeliefs in social reconstruction with ex-combatants (SoRS)**  **[Poisson GLMM; R^2^ = .15/.73; dispersion = 1.0, *p* = .504]** | | | | | |
| --- | --- | --- | --- | --- | --- |
| **Count Model** |  |  |  |  |  |
| *Predictor terms* | ***ß*** | **SE** | ***CI*** | ***z*** | ***p*** |
| intercept | 3.37 | .07 | [3.23: 3.51] | 47.13 | **< .001** |
| NETfacts | .06 | .06 | [-.05: .17] | 1.09 | .274 |
| time | -.19 | .02 | [-.23: -.15] | -9.57 | **< .001** |
| trauma | .01 | .02 | [-.04: .06] | .51 | .613 |
| *Covariates* |  |  |  |  |  |
| new trauma since baseline | -.02 | .04 | [-.11: .06] | -.53 | .598 |
| perpetration of violent acts | .01 | .03 | [-.04: .06] | .25 | .799 |
| male sex | -.08 | .05 | [-.18: .02] | -1.53 | .126 |
| age | -.02 | .02 | [-.06: .03] | -.73 | .467 |
| years of education | -.07 | .03 | [-.12: -.02] | -2.76 | **.006** |
| *Interaction terms* |  |  |  |  |  |
| NETfacts : time : trauma | - | - | - | - | ns |
| NETfacts : time | - | - | - | - | ns |
| male sex |  |  |  |  |  |
| *Random terms* | **variance** | **SD** | **n** |  |  |
| participant | .06 | .25 | 200 |  |  |
| interviewer | .02 | .13 | 17 |  |  |
