# Supplementary material for: Breaking the cycles of violence with narrative exposure: Development and feasibility of NETfacts, a community-based intervention for populations living under continuous threat
Source: PLoS One. 2022 Dec 19;17(12):e0275421. doi: 10.1371/journal.pone.0275421 (PMC9762574; doi:10.1371/journal.pone.0275421)
Supplement: S6 Table — A. GLMMs summary of the final model for IRMA. B. Post hoc Tukey tests for IRMA. (DOCX) [file pone.0275421.s009.docx]

# **Supporting information**

**S6A Table. GLMMs summary of the final model for IRMA.**

| **Rape Myths Acceptance (IRMA)**  **[Poisson GLMM; R^2^ = .07/.6; dispersion = 1.1, *p* = .112]** | | | | | |
| --- | --- | --- | --- | --- | --- |
| **Count Model** |  |  |  |  |  |
| *Predictor terms* | ***ß*** | **SE** | ***CI*** | ***z*** | ***p*** |
| intercept | 3.41 | .06 | [3.29: 3.53] | 56.59 | **< .001** |
| NETfacts | .01 | .05 | [-.08: .10] | .14 | .886 |
| time | .01 | .04 | [-.09: .09] | .18 | .854 |
| trauma | .03 | .05 | [-.07: .13] | .61 | .543 |
| *Covariates* |  |  |  |  |  |
| new trauma since baseline | .04 | .03 | [-.01: .10] | 1.54 | .124 |
| perpetration of violent acts | .02 | .02 | [-.02: .05] | 1.04 | .298 |
| male sex | .08 | .04 | [.01: .15] | 2.24 | **.025** |
| age | .01 | .02 | [-.02: .04] | .57 | .566 |
| years of education | -.05 | .02 | [-.08: -.01] | -2.72 | **.007** |
| *Interaction terms* |  |  |  |  |  |
| NETfacts : time : trauma | -.15 | .05 | [-.25: -.04] | -2.79 | **.005** |
| NETfacts : time | .00 | .05 | [-.09: .09] | .07 | .941 |
| male sex |  |  |  |  |  |
| *Random terms* | **variance** | **SD** | **n** |  |  |
| participant | .02 | .15 | 200 |  |  |
| interviewer | .02 | .14 | 17 |  |  |

**S6B Table. *Post hoc* Tukey tests for IRMA**. Within- and between-group differences in IRMA at baseline and post follow up are indicated for no direct vs direct involvement (N = 200). Tests calculated based on model predictions are marked with a number sign.

|  | | ***n_nodirect_*** | ***n_direct_*** | ***estimate*** | | ***SE*** | ***z*** | ***p*** |
| --- | --- | --- | --- | --- | --- | --- | --- | --- |
| # Number of traumatic events = 1 | | 0 | 4 |  | |  |  |  |
| *WG* | No direct involvement T0 vs T1 |  |  | 4.79 | | 2.56 | 1.87 | .241 |
|  | Direct involvement T0 vs T1 |  |  | -3.45 | | 1.44 | -2.39 | .078 |
| *BG* | T0 no direct vs direct involvement |  |  | -.84 | | 2.81 | -.30 | .991 |
|  | T1 no direct vs direct involvement |  |  | -3.45 | | 1.44 | -2.39 | .078 |
| Number of traumatic events = 2 | | 1 | 1 |  | |  |  |  |
| *WG* | No direct involvement T0 vs T1 |  |  | 4.39 | 2.38 | | 1.85 | .252 |
|  | Direct involvement T0 vs T1 |  |  | -3.16 | 1.34 | | -2.36 | .084 |
| *BG* | T0 no direct vs direct involvement |  |  | -.79 | | 2.60 | -.30 | .990 |
|  | T1 no direct vs direct involvement |  |  | -8.33 | | 2.43 | -3.43 | .003 |
| # Number of traumatic events = 3 | | 0 | 1 |  | |  |  |  |
| *WG* | No direct involvement T0 vs T1 |  |  | 3.97 | 2.19 | | 1.81 | .269 |
|  | Direct involvement T0 vs T1 |  |  | -2.87 | 1.24 | | -2.32 | .093 |
| *BG* | T0 no direct vs direct involvement |  |  | -.73 | | 2.39 | -.31 | .990 |
|  | T1 no direct vs direct involvement |  |  | -7.57 | | 2.26 | -3.34 | .005 |
| Number of traumatic events = 4 | | 2 | 8 |  | |  |  |  |
| *WG* | No direct involvement T0 vs T1 |  |  | 3.54 | 2.01 | | 1.76 | .295 |
|  | Direct involvement T0 vs T1 |  |  | -2.58 | 1.14 | | -2.27 | .106 |
| *BG* | T0 no direct vs direct involvement |  |  | -.67 | | 2.20 | -.31 | .990 |
|  | T1 no direct vs direct involvement |  |  | -6.79 | | 2.10 | -3.24 | .007 |
| Number of traumatic events = 5 | | 2 | 5 |  | |  |  |  |
| *WG* | No direct involvement T0 vs T1 |  |  | 3.09 | 1.84 | | 1.68 | .335 |
|  | Direct involvement T0 vs T1 |  |  | -2.30 | 1.05 | | -2.19 | .126 |
| *BG* | T0 no direct vs direct involvement |  |  | -.62 | | 2.01 | -.31 | .990 |
|  | T1 no direct vs direct involvement |  |  | -6.01 | | 1.94 | -3.10 | .011 |
| Number of traumatic events = 6 | | 2 | 7 |  | |  |  |  |
| *WG* | No direct involvement T0 vs T1 |  |  | 2.64 | 1.68 | | 1.57 | .397 |
|  | Direct involvement T0 vs T1 |  |  | -2.01 | .96 | | -2.09 | .156 |
| *BG* | T0 no direct vs direct involvement |  |  | -.56 | | 1.84 | -.30 | .990 |
|  | T1 no direct vs direct involvement |  |  | -5.21 | | 1.79 | -2.91 | .019 |
| Number of traumatic events = 7 | | 6 | 8 |  | |  |  |  |
| *WG* | No direct involvement T0 vs T1 |  |  | 2.17 | 1.54 | | 1.41 | .493 |
|  | Direct involvement T0 vs T1 |  |  | -1.73 | .88 | | -1.96 | .205 |
| *BG* | T0 no direct vs direct involvement |  |  | -.50 | | 1.69 | -.30 | .991 |
|  | T1 no direct vs direct involvement |  |  | -4.40 | | 1.66 | -2.65 | .040 |

|  | | ***n_nodirect_*** | ***n_direct_*** | ***estimate*** | | ***SE*** | ***z*** | ***p*** |
| --- | --- | --- | --- | --- | --- | --- | --- | --- |
| Number of traumatic events = 8 | | 6 | 13 |  | |  |  |  |
| *WG* | No direct involvement T0 vs T1 |  |  | 1.69 | 1.42 | | 1.19 | .634 |
|  | Direct involvement T0 vs T1 |  |  | -1.44 | .81 | | -1.78 | .285 |
| *BG* | T0 no direct vs direct involvement |  |  | -.44 | | 1.57 | -.28 | .992 |
|  | T1 no direct vs direct involvement |  |  | -3.57 | | 1.55 | -2.31 | .095 |
| Number of traumatic events = 9 | | 1 | 9 |  | |  |  |  |
| *WG* | No direct involvement T0 vs T1 |  |  | 1.20 | 1.34 | | .89 | .809 |
|  | Direct involvement T0 vs T1 |  |  | -1.16 | .75 | | -1.54 | .414 |
| *BG* | T0 no direct vs direct involvement |  |  | -.38 | | 1.48 | -.26 | .994 |
|  | T1 no direct vs direct involvement |  |  | -2.74 | | 1.47 | -1.87 | .243 |
| Number of traumatic events = 10 | | 4 | 20 |  | |  |  |  |
| *WG* | No direct involvement T0 vs T1 |  |  | .69 | 1.31 | | .53 | .952 |
|  | Direct involvement T0 vs T1 |  |  | -.88 | .71 | | -1.24 | .603 |
| *BG* | T0 no direct vs direct involvement |  |  | -.32 | | 1.44 | -.22 | .996 |
|  | T1 no direct vs direct involvement |  |  | -1.89 | | 1.43 | -1.32 | .55 |
| Number of traumatic events = 11 | | 2 | 6 |  | |  |  |  |
| *WG* | No direct involvement T0 vs T1 |  |  | .17 | 1.32 | | .13 | .999 |
|  | Direct involvement T0 vs T1 |  |  | -.59 | .68 | | -.87 | .818 |
| *BG* | T0 no direct vs direct involvement |  |  | -.26 | | 1.45 | -.18 | .998 |
|  | T1 no direct vs direct involvement |  |  | -1.02 | | 1.45 | -.71 | .894 |
| Number of traumatic events = 12 | | 3 | 12 |  | |  |  |  |
| *WG* | No direct involvement T0 vs T1 |  |  | -.36 | 1.40 | | -.26 | .994 |
|  | Direct involvement T0 vs T1 |  |  | -.31 | .67 | | -.47 | .966 |
| *BG* | T0 no direct vs direct involvement |  |  | -.20 | | 1.51 | -.13 | .999 |
|  | T1 no direct vs direct involvement |  |  | -.15 | | 1.51 | -.10 | 1.00 |
| Number of traumatic events = 13 | | 2 | 9 |  | |  |  |  |
| *WG* | No direct involvement T0 vs T1 |  |  | -.91 | 1.52 | | -.60 | .932 |
|  | Direct involvement T0 vs T1 |  |  | -.03 | .68 | | -.04 | 1.00 |
| *BG* | T0 no direct vs direct involvement |  |  | -.14 | | 1.62 | -.09 | 1.00 |
|  | T1 no direct vs direct involvement |  |  | .74 | | 1.64 | .45 | .969 |
| Number of traumatic events = 14 | | 4 | 6 |  | |  |  |  |
| *WG* | No direct involvement T0 vs T1 |  |  | -1.48 | 1.69 | | -.87 | .819 |
|  | Direct involvement T0 vs T1 |  |  | .25 | .70 | | .36 | .984 |
| *BG* | T0 no direct vs direct involvement |  |  | -.08 | | 1.77 | -.04 | 1.00 |
|  | T1 no direct vs direct involvement |  |  | 1.65 | | 1.81 | .91 | .798 |
| Number of traumatic events = 15 | | 2 | 8 |  | |  |  |  |
| *WG* | No direct involvement T0 vs T1 |  |  | -2.06 | 1.90 | | -1.08 | .701 |
|  | Direct involvement T0 vs T1 |  |  | .53 | .75 | | .71 | .893 |
| *BG* | T0 no direct vs direct involvement |  |  | -.02 | | 1.94 | -.01 | 1.00 |
|  | T1 no direct vs direct involvement |  |  | 2.57 | | 2.02 | 1.27 | .580 |

|  | | ***n_nodirect_*** | ***n_direct_*** | ***estimate*** | | ***SE*** | ***z*** | ***p*** |
| --- | --- | --- | --- | --- | --- | --- | --- | --- |
| # Number of traumatic events = 16 | | 0 | 9 |  | |  |  |  |
| *WG* | No direct involvement T0 vs T1 |  |  | -2.65 | 2.14 | | -1.24 | .601 |
|  | Direct involvement T0 vs T1 |  |  | .81 | .80 | | 1.01 | .744 |
| *BG* | T0 no direct vs direct involvement |  |  | .05 | | 2.14 | .02 | 1.00 |
|  | T1 no direct vs direct involvement |  |  | 3.51 | | 2.26 | 1.55 | .408 |
| # Number of traumatic events = 17 | | 0 | 5 |  | |  |  |  |
| *WG* | No direct involvement T0 vs T1 |  |  | -3.26 | 2.40 | | -1.36 | .525 |
|  | Direct involvement T0 vs T1 |  |  | 1.09 | .87 | | 1.25 | .594 |
| *BG* | T0 no direct vs direct involvement |  |  | .11 | | 2.37 | .05 | 1.00 |
|  | T1 no direct vs direct involvement |  |  | 4.46 | | 2.54 | 1.76 | .295 |
| # Number of traumatic events = 18 | | 0 | 4 |  | |  |  |  |
| *WG* | No direct involvement T0 vs T1 |  |  | -3.89 | 2.68 | | -1.45 | .468 |
|  | Direct involvement T0 vs T1 |  |  | 1.37 | .95 | | 1.44 | .472 |
| *BG* | T0 no direct vs direct involvement |  |  | .17 | | 2.60 | .07 | 1.00 |
|  | T1 no direct vs direct involvement |  |  | 5.43 | | 2.84 | 1.91 | .224 |
| # Number of traumatic events = 19 | | 0 | 3 |  | |  |  |  |
| *WG* | No direct involvement T0 vs T1 |  |  | -4.53 | 2.98 | | -1.52 | .426 |
|  | Direct involvement T0 vs T1 |  |  | 1.64 | 1.03 | | 1.60 | .380 |
| *BG* | T0 no direct vs direct involvement |  |  | .24 | | 2.85 | .08 | 1.00 |
|  | T1 no direct vs direct involvement |  |  | 6.41 | | 3.17 | 2.02 | .179 |
| Number of traumatic events = 20 | | 1 | 6 |  | |  |  |  |
| *WG* | No direct involvement T0 vs T1 |  |  | -5.19 | 3.30 | | -1.57 | .394 |
|  | Direct involvement T0 vs T1 |  |  | 1.92 | 1.12 | | 1.72 | .314 |
| *BG* | T0 no direct vs direct involvement |  |  | .30 | | 3.11 | .10 | 1.00 |
|  | T1 no direct vs direct involvement |  |  | 7.41 | | 3.51 | 2.11 | .150 |
| Number of traumatic events = 21 | | 1 | 5 |  | |  |  |  |
| *WG* | No direct involvement T0 vs T1 |  |  | -5.86 | 3.63 | | -1.62 | .370 |
|  | Direct involvement T0 vs T1 |  |  | 2.20 | 1.21 | | 1.82 | .266 |
| *BG* | T0 no direct vs direct involvement |  |  | .368 | | 3.38 | .11 | 1.00 |
|  | T1 no direct vs direct involvement |  |  | 8.43 | | 3.88 | 2.17 | .131 |
| # Number of traumatic events = 22 | | 0 | 2 |  | |  |  |  |
| *WG* | No direct involvement T0 vs T1 |  |  | -6.56 | 3.98 | | -1.65 | .352 |
|  | Direct involvement T0 vs T1 |  |  | 2.47 | 1.30 | | 1.90 | .230 |
| *BG* | T0 no direct vs direct involvement |  |  | .43 | | 3.65 | .12 | .999 |
|  | T1 no direct vs direct involvement |  |  | 9.46 | | 4.27 | 2.22 | .118 |
| # Number of traumatic events = 23 | | 0 | 1 |  | |  |  |  |
| *WG* | No direct involvement T0 vs T1 |  |  | -7.27 | 4.34 | | -1.67 | .338 |
|  | Direct involvement T0 vs T1 |  |  | 2.75 | 1.40 | | 1.96 | .204 |
| *BG* | T0 no direct vs direct involvement |  |  | .50 | | 3.94 | .13 | .999 |
|  | T1 no direct vs direct involvement |  |  | 10.51 | | 4.67 | 2.25 | .110 |

|  | | ***n_nodirect_*** | ***n_direct_*** | ***estimate*** | | ***SE*** | | ***z*** | ***p*** |
| --- | --- | --- | --- | --- | --- | --- | --- | --- | --- |
| # Number of traumatic events = 24 | | 1 | 0 |  | |  | |  |  |
| *WG* | No direct involvement T0 vs T1 |  |  | -8.00 | 4.72 | | -1.69 | | .327 |
|  | Direct involvement T0 vs T1 |  |  | 3.02 | 1.50 | | 2.01 | | .184 |
| *BG* | T0 no direct vs direct involvement |  |  | .57 | | 4.22 | | .13 | .999 |
|  | T1 no direct vs direct involvement |  |  | 11.59 | | 5.10 | | 2.27 | .104 |
| # Number of traumatic events = 25 | | 0 | 1 |  | |  | |  |  |
| *WG* | No direct involvement T0 vs T1 |  |  | -8.75 | 5.12 | | -1.71 | | .319 |
|  | Direct involvement T0 vs T1 |  |  | 3.29 | 1.60 | | 2.05 | | .169 |
| *BG* | T0 no direct vs direct involvement |  |  | .64 | | 4.52 | | .14 | .999 |
|  | T1 no direct vs direct involvement |  |  | 12.68 | | 5.54 | | 2.29 | .101 |
| # Number of traumatic events = 26 | | 0 | 1 |  | |  | |  |  |
| *WG* | No direct involvement T0 vs T1 |  |  | -9.51 | 5.53 | | -1.72 | | .313 |
|  | Direct involvement T0 vs T1 |  |  | 3.57 | 1.71 | | 2.09 | | .156 |
| *BG* | T0 no direct vs direct involvement |  |  | .70 | | 4.82 | | .15 | .999 |
|  | T1 no direct vs direct involvement |  |  | 13.78 | | 6.00 | | 2.30 | .099 |
| # Number of traumatic events = 27 | | 0 | 0 |  | |  | |  |  |
| *WG* | No direct involvement T0 vs T1 |  |  | -10.30 | 5.95 | | -1.73 | | .308 |
|  | Direct involvement T0 vs T1 |  |  | 3.84 | 1.81 | | 2.12 | | .147 |
| *BG* | T0 no direct vs direct involvement |  |  | .77 | | 5.12 | | .15 | .999 |
|  | T1 no direct vs direct involvement |  |  | 14.91 | | 6.48 | | 2.30 | .098 |
| # Number of traumatic events = 28 | | 0 | 3 |  | |  | |  |  |
| *WG* | No direct involvement T0 vs T1 |  |  | -11.11 | 6.40 | | -1.74 | | .304 |
|  | Direct involvement T0 vs T1 |  |  | 4.11 | 1.92 | | 2.15 | | .139 |
| *BG* | T0 no direct vs direct involvement |  |  | .84 | | 5.43 | | .16 | .999 |
|  | T1 no direct vs direct involvement |  |  | 16.06 | | 6.99 | | 2.30 | .098 |
| # Number of traumatic events = 29 | | 0 | 2 |  | |  | |  |  |
| *WG* | No direct involvement T0 vs T1 |  |  | -11.94 | 6.85 | | -1.74 | | .302 |
|  | Direct involvement T0 vs T1 |  |  | 4.38 | 2.02 | | 2.17 | | .133 |
| *BG* | T0 no direct vs direct involvement |  |  | .91 | | 5.74 | | .16 | .999 |
|  | T1 no direct vs direct involvement |  |  | 17.23 | | 7.51 | | 2.30 | .099 |
| # Number of traumatic events = 30 | | 0 | 1 |  | |  | |  |  |
| *WG* | No direct involvement T0 vs T1 |  |  | -12.79 | 7.33 | | -1.75 | | .301 |
|  | Direct involvement T0 vs T1 |  |  | 4.65 | 2.13 | | 2.18 | | .128 |
| *BG* | T0 no direct vs direct involvement |  |  | .98 | | 6.05 | | .16 | .999 |
|  | T1 no direct vs direct involvement |  |  | 18.42 | | 8.05 | | 2.29 | .101 |
