# Supplementary material for: Breaking the cycles of violence with narrative exposure: Development and feasibility of NETfacts, a community-based intervention for populations living under continuous threat
Source: PLoS One. 2022 Dec 19;17(12):e0275421. doi: 10.1371/journal.pone.0275421 (PMC9762574; doi:10.1371/journal.pone.0275421)
Supplement: S7 Table — (DOCX) [file pone.0275421.s010.docx]

# **Supporting information**

**S7 Table. GLMMs summary of the final model for SAQ.**

| **Perceived general social acknowledgement as trauma survivor (SAQ)**  **[Zero-inflated truncated Poisson GLMM; R^2^ = .23/.9; dispersion = .96, *p* = .776]** | | | | | |
| --- | --- | --- | --- | --- | --- |
| **Count Model** |  |  |  |  |  |
| *Predictor terms* | ***ß*** | **SE** | ***CI*** | ***z*** | ***p*** |
| intercept | 1.39 | .16 | [1.08: 1.70] | 8.88 | **< .001** |
| NETfacts | .07 | .12 | [-.17: .31] | .58 | .561 |
| time | .13 | .06 | [.01: .26] | 2.11 | **.035** |
| trauma | .19 | .05 | [.09: .29] | 3.70 | **< .001** |
| *Covariates* |  |  |  |  |  |
| new trauma since baseline | .02 | .09 | [-.15: .19] | .26 | .796 |
| perpetration of violent acts | -.02 | .05 | [-.13: .08] | -.47 | .638 |
| male sex | -.03 | .11 | [-.24: .19] | -.26 | .795 |
| age | -.00 | .05 | [-.09: .09] | -.04 | .972 |
| years of education | -.15 | .06 | [-.26: -.05] | -2.80 | .005 |
| *Interaction terms* |  |  |  |  |  |
| NETfacts : time : trauma | - | - | - | - | ns |
| NETfacts : time | - | - | - | - | ns |
| **Zero-Inflated Model** |  |  |  |  |  |
| *Predictor terms* |  |  |  |  |  |
| intercept | -.67 | .46 | [-1.57: .24] | -1.42 | .154 |
| NETfacts | -.01 | .33 | [-.66: .64] | -.03 | .977 |
| time | -.81 | .26 | [-1.32: -.30] | -3.13 | **.002** |
| trauma | -.63 | .17 | [-.97: -.29] | -3.67 | **<.001** |
| *Covariates* |  |  |  |  |  |
| new trauma since baseline | -.34 | .27 | [-.88: .19] | -1.27 | .206 |
| perpetration of violent acts | .17 | .17 | [-.18: .52] | .96 | .340 |
| male sex | .03 | .31 | [-.59: .64] | .08 | .933 |
| age | .01 | .14 | [-.26: .29] | .10 | .923 |
| years of education | .22 | .17 | [-.10: .54] | 1.34 | .179 |
| *Interaction terms* |  |  |  |  |  |
| NETfacts : time : trauma | - | - | - | - | ns |
| NETfacts : time | - | - | - | - | ns |
| *Random terms* | **variance** | **SD** | **n** |  |  |
| participant | .13 | .37 | 189 |  |  |
| interviewer | .07 | .27 | 17 |  |  |
